# Endothelial deletion of PTBP1 disrupts ventricular chamber development

**DOI:** 10.1038/s41467-023-37409-9

**Published:** 2023-03-31

**Authors:** Hongyu Liu, Ran Duan, Xiaoyu He, Jincu Qi, Tianming Xing, Yahan Wu, Liping Zhou, Lingling Wang, Yujing Shao, Fulei Zhang, Huixing Zhou, Xingdong Gu, Bowen Lin, Yuanyuan Liu, Yan Wang, Yi Liu, Li Li, Dandan Liang, Yi-Han Chen

**Affiliations:** 1 Department of Cardiology, Shanghai East Hospital, Tongji University School of Medicine, 200120 Shanghai, China; 2 Key Laboratory of Arrhythmias of the Ministry of Education of China, Shanghai East Hospital, Tongji University School of Medicine, 200120 Shanghai, China; 3 Jinzhou Medical University, 121000 Jinzhou, Liaoning China; 4 Research Units of Origin and Regulation of Heart Rhythm, Chinese Academy of Medical Sciences, 200092 Shanghai, China; 5 Department of Pathology and Pathophysiology, Tongji University School of Medicine, 200092 Shanghai, China

**Keywords:** Heart development, Cell migration, Alternative splicing, Organogenesis

## Abstract

The growth and maturation of the ventricular chamber require spatiotemporally precise synergy between diverse cell types. Alternative splicing deeply affects the processes. However, the functional properties of alternative splicing in cardiac development are largely unknown. Our study reveals that an alternative splicing factor polypyrimidine tract-binding protein 1 (PTBP1) plays a key role in ventricular chamber morphogenesis. During heart development, PTBP1 colocalizes with endothelial cells but is almost undetectable in cardiomyocytes. The endothelial-specific knockout of *Ptbp1*, in either endocardial cells or pan-endothelial cells, leads to a typical phenotype of left ventricular noncompaction (LVNC). Mechanistically, the deletion of *Ptbp1* reduces the migration of endothelial cells, disrupting cardiomyocyte proliferation and ultimately leading to the LVNC. Further study shows that *Ptbp1* deficiency changes the alternative splicing of β-arrestin-1 (*Arrb1*), which affects endothelial cell migration. In conclusion, as an alternative splicing factor, PTBP1 is essential during ventricular chamber development, and its deficiency can lead to congenital heart disease.

## Abstract (web summary)

Alternative splicing crucially affects various biological processes, however, its function in heart development is largely unknown. Here, the authors show an essential role of alternative splicing factor PTBP1 in ventricular chamber development.

## Introduction

During mammalian heart development, compaction of the trabecular myocardium is crucial to ventricular chamber morphogenesis^1,2^. At the early developmental stage, the formation of trabeculae facilitates oxygen uptake and nutrient supply. As development proceeds, this function of trabeculae is gradually degraded and is replaced by coronary blood vessels. The majority of trabeculae then undergo compaction, contributing to the formation of a mature ventricular chamber^3,4^. Defective trabecular compaction often manifests as left ventricular noncompaction (LVNC), characterized by an overgrown trabecular myocardium, a thin compact myocardium and deep intertrabecular recesses^5,6^. As the third most common cardiomyopathy after dilated and hypertrophic cardiomyopathy, LVNC is increasingly recognized as an important cause of systemic embolism, malignant arrhythmia, heart failure, and sudden death^7–9^.

Ventricular trabeculation and compaction are dynamic processes governed by interactions between distinct cell types. The precise coordination of endothelial cells and cardiomyocytes has been demonstrated to be critical for ventricular growth and trabeculation^10–12^. Several pathways, including Notch and Neuregulin signaling, have been identified as key regulators of these processes and are tightly associated with the pathogenesis of LVNC^13–16^. However, there is still a lack of sufficient knowledge on the control of normal trabeculation and compaction during ventricular development. Elucidation of the underlying mechanism remains a challenge in the field of heart development. Alternative splicing is an important posttranscriptional regulation that crucially affects various biological processes^17–19^. As an alternative splicing factor, polypyrimidine tract-binding protein 1 (PTBP1) controls numerous alternative splicing events^20,21^. The homozygous mutation in *Ptbp1* is reported to severely affect the proliferation of embryonic stem cells, leading to embryonic lethality in the early developmental stage^22^.

Here, we reveal that PTBP1 characteristically colocalizes with endothelial cells during ventricular chamber development. Endothelial-specific knockout of *Ptbp1* leads to LVNC by regulating the migration of endothelial cells and the proliferation of cardiomyocytes. The switching change between the expression ratio of two ARRB1 isoforms is observed in *Ptbp1*-deficient endothelial cells and is shown to affect endothelial cell migration. Furthermore, the splicing events and altered gene expression caused by *Ptbp1* deficiency are involved in biological processes and signaling pathways tightly associated with cardiac developmental defects, including LVNC.

## Results

### PTBP1 colocalizes with endothelial cells during heart development

**Fig. 1:**
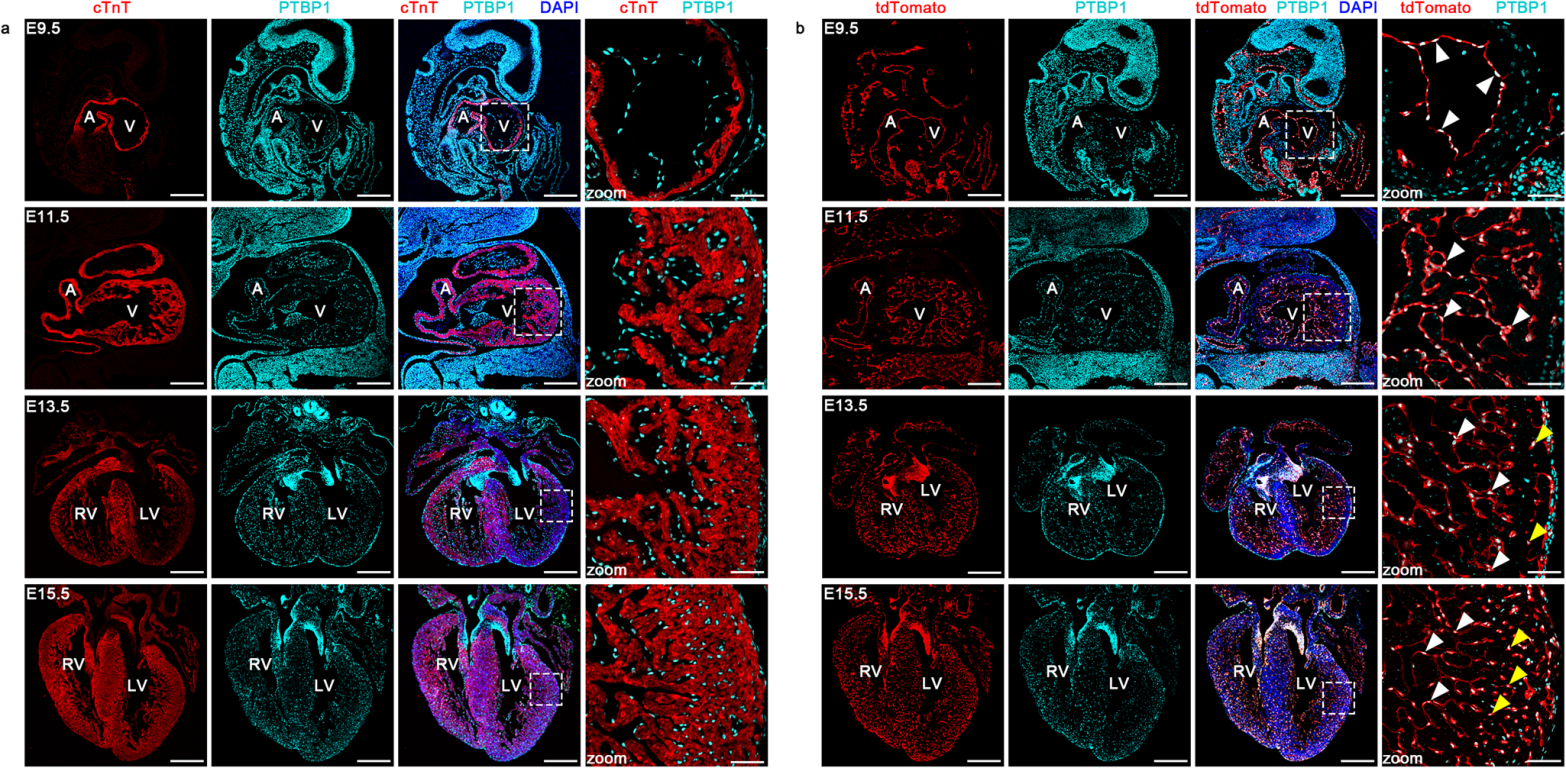
The colocalization of PTBP1 with endothelial cells during heart development. **a** Immunofluorescence staining of PTBP1 (cyan) with the cardiomyocyte marker cTnT (red) in embryonic hearts. The magnified images indicate the distribution pattern of PTBP1 in ventricular tissues. The expression of PTBP1 is almost undetectable in cardiomyocytes. **b** Immunofluorescence staining of PTBP1 in *Tie2-Cre*;*Rosa26*-tdTomato embryonic hearts. The magnified images indicate the colocalization of PTBP1 (cyan) with tdTomato-marked endothelial cells (red) in *Tie2-Cre*;*Rosa26*-tdTomato ventricles. The white arrowheads indicate the colocalization of PTBP1 with endocardial cells; the yellow arrowheads indicate the colocalization of PTBP1 with endothelial cells in the compact myocardium. Scale bars in E9.5 and E11.5 images are 200 µm; Scale bars in E13.5 and E15.5 images are 400 µm; Scale bars in the zoomed images are 50 µm. A atrium, V ventricle, LV left ventricle, RV right ventricle.

To identify the expression pattern of PTBP1 in the embryonic heart, we examined its localization at different embryonic stages utilizing the genetic lineage tracing system in combination with immunofluorescence staining. The co-staining of PTBP1 with the myocardial marker cTnT demonstrated that PTBP1 was rarely localized within the myocardium, while enriched expression was observed in the endocardium and endocardial cushions at the stage of embryonic day (E) 9.5 and E11.5 (Fig. 1a). We then performed colocalization analysis of PTBP1 and endothelial cells using the *Tie2-Cre*;*Rosa26*-tdTomato genetic lineage tracing system. At E9.5, the innermost layer of the ventricular wall is composed of endocardial cells, which are specialized endothelial cells^10,23^. We observed a significant overlap of PTBP1 with tdTomato, indicating that PTBP1 colocalized with endocardial cells at this stage (Fig. 1b). As the trabeculae extended, PTBP1 expression was also detected in the endocardial cells of the trabecular muscle at E11.5, along with the formation of the trabecular myocardium (Fig. 1b).

From E13.5, the ventricular wall dramatically thickens and gradually grows into a mature chamber^14^. The expression of PTBP1 was observed in the compact myocardium at this stage but was predominantly localized in endothelial cells instead of cardiomyocytes (Fig. 1a, b). In addition, the characteristic expression of PTBP1 was also confirmed by immunofluorescence staining with the endothelial cell marker VE-cadherin (Supplementary Fig. 1). Collectively, these data revealed the distinct localization of PTBP1 in the embryonic heart, which was accompanied with the migration of endothelial cells during ventricular morphogenesis.

### Endothelial-specific deletion of *Ptbp1* in mice leads to LVNC

**Fig. 2:**
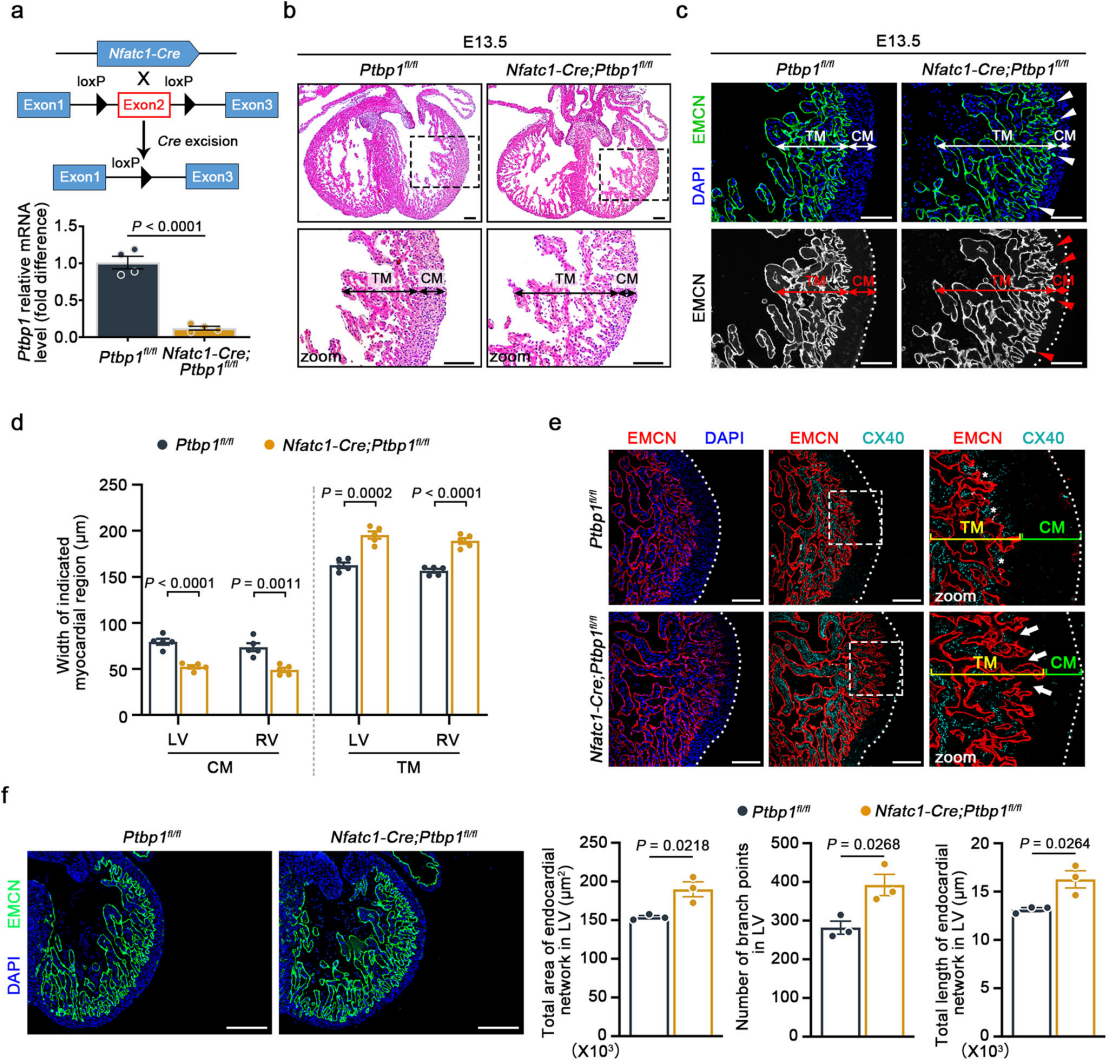
Endocardial-specific knockout of *Ptbp1* leads to ventricular noncompaction. **a** Generation of the *Ptbp1* endocardial-specific knockout (*Nfatc1-Cre*;*Ptbp1*^*fl/fl*^) mouse line. Top: schematic diagram showing the generation of the *Nfatc1-Cre*;*Ptbp1*^*fl/fl*^ mouse line. Bottom: qRT-PCR examination of the *Ptbp1* mRNA level in endothelial cells isolated from E13.5 *Ptbp1*^*fl*/*fl*^ and *Nfatc1-Cre*;*Ptbp1*^*fl/fl*^ hearts. *n* = 4 mice per group. **b** H&E staining showing the trabecular myocardium (TM) and compact myocardium (CM) in E13.5 *Ptbp1*^*fl*/*fl*^ and *Nfatc1-Cre*;*Ptbp1*^*fl/fl*^ heart tissue sections. Scale bars, 100 µm. **c**, **d** Representative images and quantification of the TM and CM thickness in E13.5 *Ptbp1*^*fl*/*fl*^ and *Nfatc1-Cre*;*Ptbp1*^*fl/fl*^ hearts. The graph shows the reduced thickness of the CM and increased thickness of the TM in *Nfatc1-Cre*;*Ptbp1*^*fl/fl*^ hearts. *n* = 5 mice per group. The arrowheads indicate the expression of EMCN in the *Nfatc1-Cre*;*Ptbp1*^*fl/fl*^ hearts. LV left ventricle, RV right ventricle. Scale bars, 100 µm. **e** Co-immunofluorescence staining of the endocardial cell marker EMCN and the trabecular myocardium marker CX40 in E13.5 *Ptbp1*^*fl*/*fl*^ and *Nfatc1-Cre*;*Ptbp1*^*fl/fl*^ heart sections. Scale bars, 100 µm. The asterisks indicate the expression of CX40 in the *Ptbp1*^*fl/fl*^ hearts. The arrows indicate the reduced expression of CX40 in the *Nfatc1-Cre*;*Ptbp1*^*fl/fl*^ hearts. **f** Immunofluorescence staining of the endomucin (EMCN) (green) in E13.5 *Nfatc1-Cre*;*Ptbp1*^*fl*/*fl*^ and *Ptbp1*^*fl*/*fl*^ heart sections. Scale bars, 200 µm. The graphs show quantification of the number of endocardial branch points, total area covered by the endocardial network, and total length of the endocardial network of E13.5 *Nfatc1-Cre*;*Ptbp1*^*fl*/*fl*^ and *Ptbp1*^*fl*/*fl*^ hearts. *n* = 3 mice per group. The data are presented as the mean ± s.e.m. *P* values were calculated by unpaired two-tailed *t* test. Source data are provided as a Source Data file.

Considering the characteristic localization of PTBP1, we constructed three endothelial-specific knockout mouse lines to explore its potential role in cardiac development. By crossing *Ptbp1*^flox/flox^ (*Ptbp1*^*fl*/*fl*^) mice with *Nfatc1*-*Cre* mice, we deleted *Ptbp1* in endocardial cells, which are known to support early myocardial trabeculation as well as compact myocardial growth at later developmental stages^10,15,24^. The expression of *Ptbp1* mRNA and PTBP1 protein was significantly reduced in the endothelial cells of *Nfatc1*-*Cre*;*Ptbp1*^*fl*/*fl*^ mice, indicating that the *Ptbp1*^*fl*/*fl*^ allele was effectively recombined (Fig. 2a and Supplementary Fig. 2). No significant increase in embryonic death was observed in *Nfatc1-Cre*;*Ptbp1*^*fl/fl*^ mice (Supplementary Table 1). Histological examination at E13.5 revealed a thin compact myocardium with significantly protruding trabeculae toward the ventricular lumen in *Nfatc1*-*Cre*;*Ptbp1*^*fl*/*fl*^ mice (Fig. 2b).

In addition, we performed immunofluorescence analysis with endomucin (EMCN) to better distinguish between the trabecular and compact myocardium. EMCN staining further demonstrated the decreased thickness of the compact myocardium and the expanded trabecular myocardium in *Nfatc1*-*Cre*;*Ptbp1*^*fl*/*fl*^ mice (Fig. 2c, d). Connexin 40 (CX40) is considered to be one of the markers of embryonic trabecular myocardium and is abnormally expressed in the trabecular myocardium adjacent to the thinned compact myocardium in LVNC^14,24^. The immunofluorescence staining for CX40 on embryonic heart sections showed that the abnormal trabeculae adjacent to the thin compact myocardium lacked CX40 expression in *Nfatc1*-*Cre*;*Ptbp1*^*fl*/*fl*^ mice (Fig. 2e). In addition, the abnormal extension of the endocardium to the compact myocardium was also demonstrated by EMCN staining (Fig. 2e). We also examined whether the endocardial complexity was changed after *Ptbp1* knockout in *Nfatc1-Cre*;*Ptbp1*^*fl/fl*^ mice compared to *Ptbp1*^*fl/fl*^ mice. The increased number of endocardial branch points, total area covered by the endocardial network, and total length of the endocardial network were observed in *Nfatc1-Cre*;*Ptbp1*^*fl/fl*^ hearts (Fig. 2f), suggesting that *Ptbp1* deficiency in endocardial cells affected the endocardial complexity in embryonic hearts. These data confirmed the ventricular noncompaction in *Nfatc1*-*Cre*;*Ptbp1*^*fl*/*fl*^ mice.

**Fig. 3:**
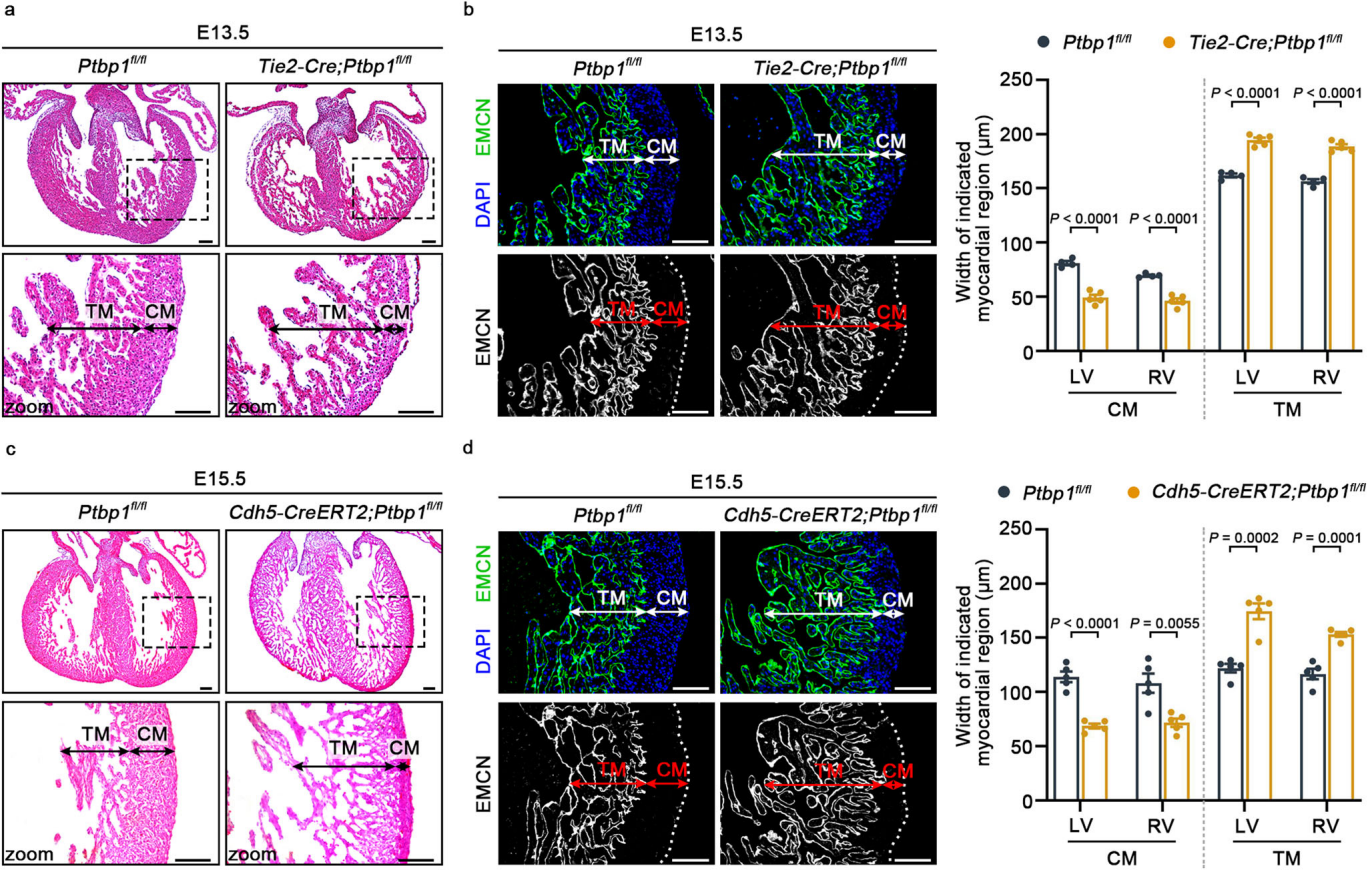
*Ptbp1* knockout in pan-endothelial cells causes ventricular noncompaction. **a** H&E staining showing the trabecular myocardium (TM) and compact myocardium (CM) in E13.5 *Ptbp1*^*fl*/*fl*^ and *Tie2-Cre*;*Ptbp1*^*fl/fl*^ heart sections. Scale bars, 100 µm. **b** Immunofluorescence staining of endomucin (EMCN) in E13.5 *Ptbp1*^*fl*/*fl*^ and *Tie2-Cre*;*Ptbp1*^*fl/fl*^ heart sections. The graph shows the quantification of the CM and TM thickness. *n* = 4 mice for *Ptbp1*^*fl*/*fl*^ group and *n* = 5 mice for *Tie2-Cre*;*Ptbp1*^*fl/fl*^ group. Scale bars, 100 µm. **c** H&E staining shows the TM and CM in E15.5 *Ptbp1*^*fl*/*fl*^ and *Cdh5-CreERT2*;*Ptbp1*^*fl/fl*^ mice heart sections. Scale bars, 100 µm. **d** Immunofluorescence staining of EMCN in E15.5 *Ptbp1*^*fl*/*fl*^ and *Cdh5-CreERT2*;*Ptbp1*^*fl/fl*^ heart sections. The graph shows the quantification of the CM and TM thickness in E15.5 *Ptbp1*^*fl*/*fl*^ and *Cdh5-CreERT2*;*Ptbp1*^*fl/fl*^ heart sections. *n* = 5 mice per group. LV left ventricle, RV right ventricle. Scale bars, 100 µm. The data are presented as the mean ± s.e.m. *P* values were calculated by unpaired two-tailed *t* test. Source data are provided as a Source Data file.

We next used two pan-endothelial driver lines, *Tie2*-*Cre* and *Cdh5*-*CreERT2*, to investigate the role of PTBP1 in ventricular chamber morphogenesis. The reduced expression of *Ptbp1* was confirmed in cardiac endothelial cells from *Tie2*-*Cre*;*Ptbp1*^*fl*/*fl*^ and *Cdh5*-*CreERT2*;*Ptbp1*^*fl*/*fl*^ mice (Supplementary Figs. 2 and 3). The calculation of survival rates showed the embryonic death of *Cdh5-CreERT2*;*Ptbp1*^*fl/fl*^ or *Tie2-Cre*;*Ptbp1*^*fl/fl*^ mice was increased from E12.5 to E13.5 (Supplementary Tables 2 and 3). Histological analysis and EMCN staining demonstrated that the deletion of *Ptbp1* severely disrupted the ventricular development in *Tie2*-*Cre*;*Ptbp1*^*fl*/*fl*^ mice and *Cdh5*-*CreERT2*;*Ptbp1*^*fl*/*fl*^ mice, which exhibited a thickened trabecular myocardium and thinned compact myocardium, consistent with the cardiac phenotype of *Nfatc1*-*Cre*;*Ptbp1*^*fl*/*fl*^ mice (Fig. 3a–d). The CX40 staining demonstrated the ventricular noncompaction phenotype in the two mouse lines (Supplementary Fig. 4).

To determine the onset of cardiac phenotype in *Ptbp1*-deficient mice, we also performed H&E and immunofluorescence staining of EMCN at earlier stages. The thinned compact myocardium was observed at E12.5, while there were no significant changes in trabecular myocardium thickness (Supplementary Fig. 5). The increased trabecular myocardium thickness could be observed in *Ptbp1*-deficient mice at E13.5 (Figs. 2 and 3). The incidence of the LVNC phenotype was 77% (10 out of 13) in *Nfatc1-Cre*;*Ptbp1*^*fl/fl*^ embryos, 75% (6 out of 8) in *Tie2-Cre*;*Ptbp1*^*fl/fl*^ embryos, and 75% (12 out of 16) in *Cdh5-CreERT2*;*Ptbp1*^*fl/fl*^ embryos, respectively. In addition, we also examined the cardiac phenotype of heterozygous *Ptbp1*-deficient mice, which exhibited normal ventricular morphology and structure (Supplementary Fig. 6).

**Fig. 4:**
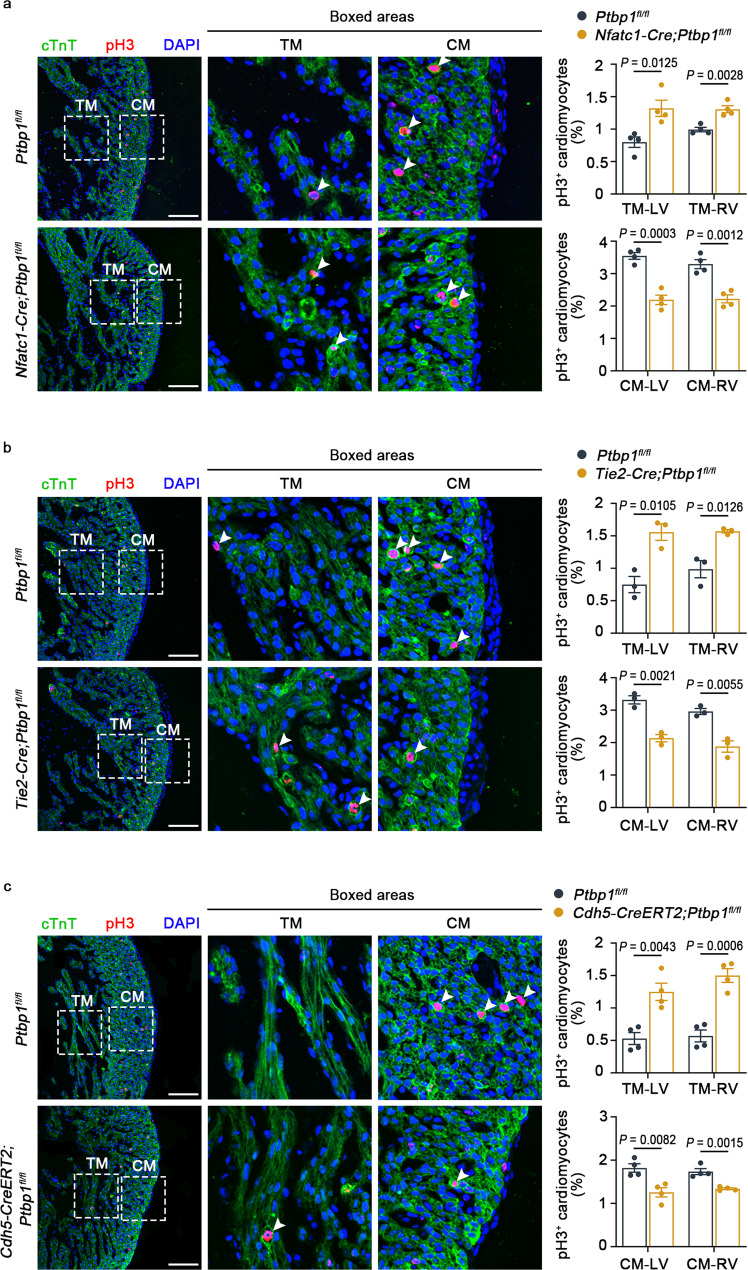
The proliferation of cardiomyocytes is disrupted in *Ptbp1* knockout mice. **a**–**c** Co-immunostaining of the cell proliferation marker pH3 (red) and cardiomyocyte marker cTnT (green) shows disrupted proliferation of cardiomyocytes in heart sections from E13.5 *Nfatc1-Cre*;*Ptbp1*^*fl/fl*^ (**a**), E13.5 *Tie2-Cre*;*Ptbp1*^*fl/fl*^ (**b**) and E15.5 *Cdh5-CreERT2*;*Ptbp1*^*fl/fl*^ (**c**) mice, respectively. *n* = 4 mice per group in (**a**), *n* = 3 mice per group in (**b**), *n* = 4 mice per group in (**c**). Scale bars, 100 µm. The graph shows the quantification of pH3^+^ cardiomyocytes in different groups. The arrowheads indicate the pH3-positive cardiomyocytes. TM trabecular myocardium, CM compact myocardium, LV left ventricle, RV right ventricle. The data are presented as the mean ± s.e.m. *P* values were calculated by unpaired two-tailed *t* test. Source data are provided as a Source Data file.

Furthermore, we examined cardiomyocyte proliferation in the trabecular and compact myocardium. Markedly decreased proliferation of compact cardiomyocytes and increased proliferation of trabecular cardiomyocytes were observed in *Nfatc1*-*Cre*;*Ptbp1*^*fl*/*fl*^, *Tie2*-*Cre*;*Ptbp1*^*fl*/*fl*^ and *Cdh5*-*CreERT2*;*Ptbp1*^*fl*/*fl*^ mice (Fig. 4a–c), demonstrating the abnormal proliferation of cardiomyocyte in *Ptbp1*-deficient mice, which is one of the features of ventricular noncompaction^25–27^. Collectively, these findings indicated that *Ptbp1* deficiency in endothelial cells affected ventricular chamber development, leading to the occurrence of LVNC.

### *Ptbp1* deletion inhibits cardiac endothelial cell migration

**Fig. 5:**
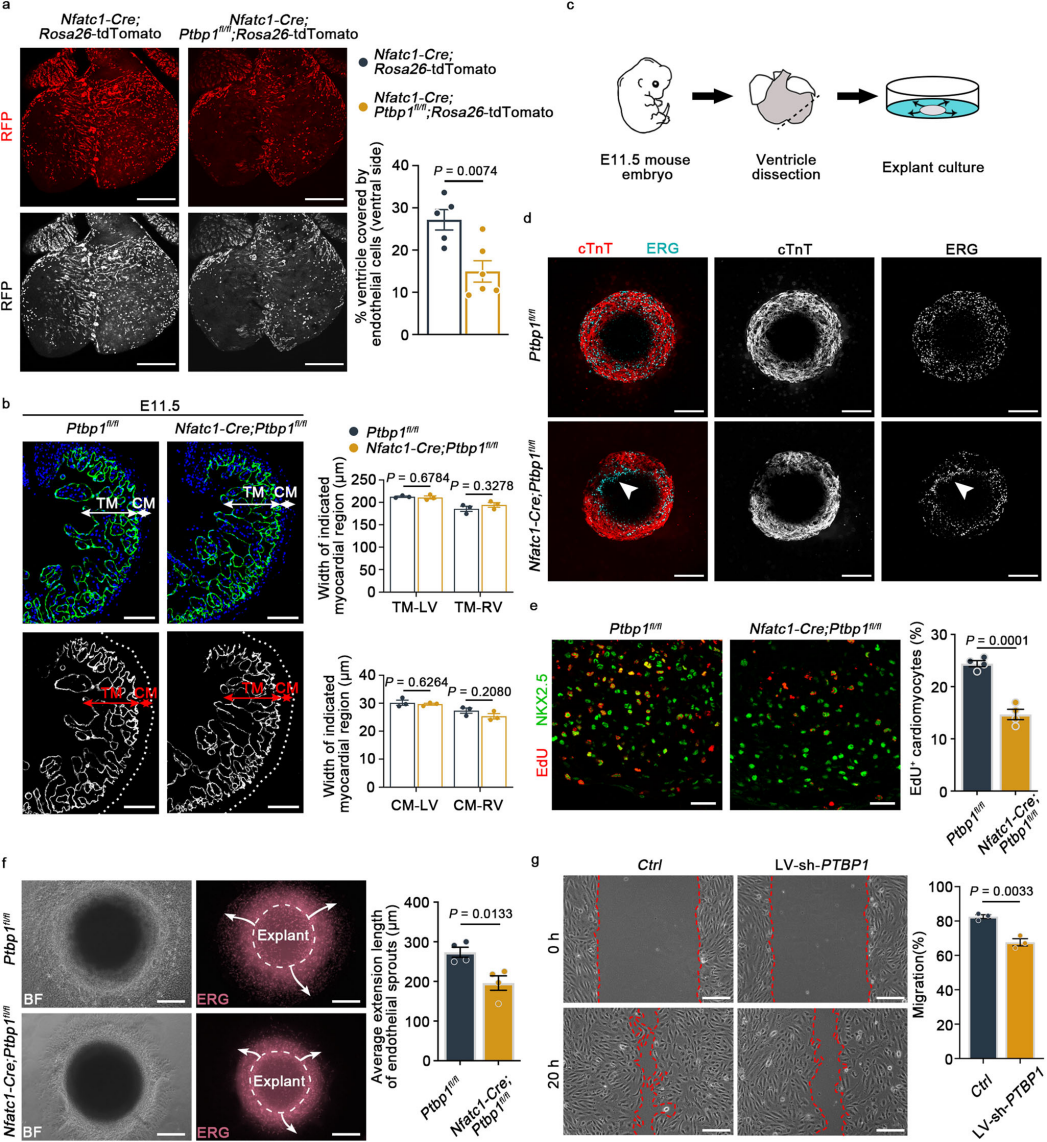
*Ptbp1* knockdown affects endothelial cell migration and cardiomyocyte proliferation. **a** Representative images and quantification showing the significantly decreased migration of endothelial cells (labeled with RFP) in E13.5 *Nfatc1-Cre*;*Ptbp1*^*fl/fl*^;*Rosa26*-tdTomato mice compared to *Nfatc1-Cre*;*Rosa26*-tdTomato mice, which were assessed by the percentage of endothelial cell coverage on the ventral side of the heart. Scale bars, 200 µm. *n* = 5 mice for *Nfatc1-Cre*;*Rosa26*-tdTomato group, *n* = 6 mice for *Nfatc1-Cre*;*Ptbp1*^*fl/fl*^;*Rosa26*-tdTomato group. **b** Immunofluorescence staining of EMCN in heart sections from E11.5 *Ptbp1*^*fl*/*fl*^ and *Nfatc1-Cre*;*Ptbp1*^*fl*/*fl*^ mice. Scale bars, 100 µm. The graph shows the thickness of the compact myocardium (CM) and trabecular myocardium (TM) of E11.5 *Ptbp1*^*fl*/*fl*^ and *Nfatc1-Cre*;*Ptbp1*^*fl*/*fl*^ mice. *n* = 3 mice per group. **c** Schematic diagram showing the experimental strategy of explant culture. **d** Immunostaining for cTnT (red) and ERG (cyan) in ventricular explants from *Ptbp1*^*fl*/*fl*^ and *Nfatc1-Cre*;*Ptbp1*^*fl/fl*^ mice after 2 days culture. Scale bars, 200 µm. The arrowheads indicate decelerated endothelial cell migration in the explanted tissues. **e** Left: immunostaining for NKX2.5 (red) and EdU (green) in *Ptbp1*^*fl*/*fl*^ and *Nfatc1-Cre*;*Ptbp1*^*fl/fl*^ ventricular explants cultured for 2 days. Scale bars, 25 µm. Right: Quantification of the EdU-positive cardiomyocytes in ventricular explants. *n* = 4 explants per group. **f** Representative images and quantification of ventricular explants showing the migration of endothelial cells (labeled with ERG) at 5 days after culture. The arrows indicate the direction of endothelial cell sprouting. Scale bars, 200 µm. *n* = 4 explants per group. **g** Representative images and quantification of the in vitro scratch assays in primary human umbilical endothelial cells (HUVECs) after *PTBP1* knockdown (LV-sh-*PTBP1*). Scale bars, 200 µm. *n* = 3 independent experiments. BF bright field, TM trabecular myocardium, CM compact myocardium, LV left ventricle, RV right ventricle, Ctrl control lentivirus-shRNA, LV-sh-P*TBP1* lentivirus-shRNA-*PTBP1*. The data are presented as the mean ± s.e.m. *P* values were calculated by unpaired two-tailed *t* test. Source data are provided as a Source Data file.

To clarify the cellular processes underlying ventricular noncompaction in *Ptbp1*-deficient mice, we first examined the proliferation and apoptosis of endocardial cells in three endothelial-specific *Ptbp1* knockout mouse lines. The results showed that they were not significantly changed in *Ptbp1* deficiency mice compared to that in *Ptbp1*^*fl/fl*^ mice (Supplementary Figs. 7 and 8). In addition, there was no significant difference in the number of endocardial cells after *Ptbp1* knockout (Supplementary Fig. 9). We then used a *Nfatc1-Cre*; *Rosa26*-tdTomato genetic lineage tracing system to specifically label endothelial cells and performed in vivo migration experiment. The whole-mount immunofluorescence staining of embryonic hearts confirmed the decreased migration of endothelial cells in *Ptbp1* deficiency mice compared to control mice (Fig. 5a). We also investigated the potential vascular defects in pan-endothelial *Ptbp1* knockout mice. The immunofluorescence staining analysis showed that the ventricular area covered by endothelial cells on the dorsal side of hearts was reduced in *Tie2-Cre*;*Ptbp1*^*fl/fl*^;*Rosa26*-tdTomato and *Cdh5-CreERT2;Ptbp1*^*fl/fl*^;*Rosa26*-tdTomato mice compared to control mice (Supplementary Fig. 10), suggesting that the coronary vessel growth was also suppressed after *Ptbp1* deletion.

We then investigated how the endothelial-specific knockout of *Ptbp1* led to abnormal cardiomyocyte proliferation using explanted ventricles from *Nfatc1*-*Cre*;*Ptbp1*^*fl*/*fl*^ mice. No obvious structural defects were observed in *Nfatc1*-*Cre*;*Ptbp1*^*fl*/*fl*^ hearts at E11.5 as determined by immunofluorescence analysis of EMCN (Fig. 5b). After 2 days of culture, decelerated endothelial cell migration was observed in explanted tissues from *Nfatc1*-*Cre*;*Ptbp1*^*fl*/*fl*^ hearts (Fig. 5c, d). The examination of cardiomyocyte proliferation in explanted ventricular tissues showed that the percentage of EdU-positive cardiomyocytes was decreased in tissues from *Nfatc1*-*Cre*;*Ptbp1*^*fl*/*fl*^ hearts (Fig. 5e).

As the culture time extended, the endothelial cells gradually migrated out of the explanted ventricular tissues. The endothelial cell migration in plated ventricular tissues from *Nfatc1*-*Cre*;*Ptbp1*^*fl*/*fl*^ mice was significantly reduced compared to that in *Ptbp1*^*fl*/*fl*^ ventricular tissues after culturing for 5 days (Fig. 5f). To confirm the effect of PTBP1 on cell migration, we also performed scratch assays with HUVECs. The migration of cells treated with lentivirus-shRNA-*PTBP1* (LV-sh-*PTBP1*) was also slowed compared with that of the controls (Fig. 5g). Collectively, these data demonstrated that *Ptbp1* deficiency affected the migration of endothelial cells, thereby reducing the proliferation of cardiomyocytes.

### PTBP1 regulates the alternative splicing of *Arrb1* in endothelial cells

**Fig. 6:**
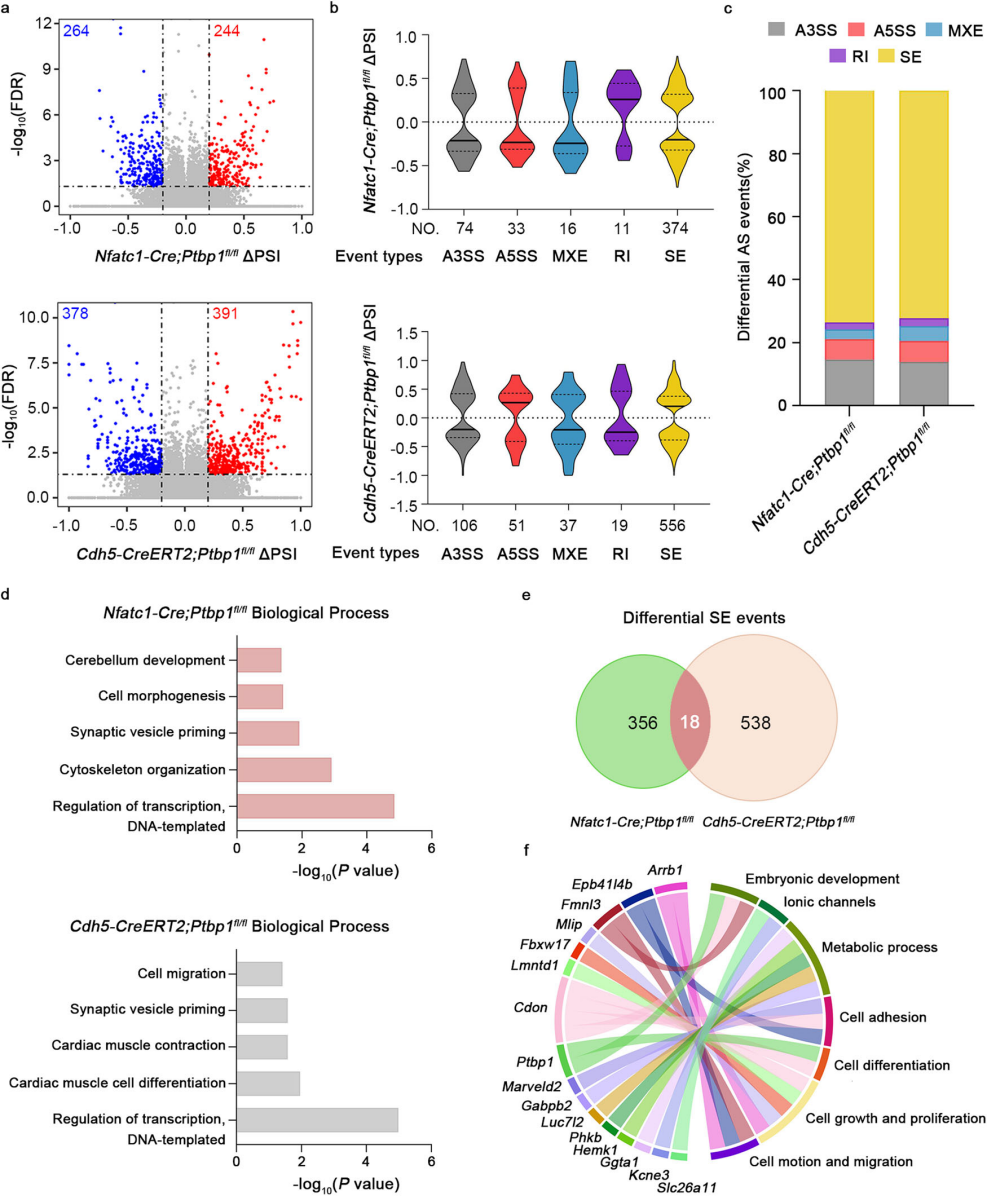
PTBP1 regulates alternative splicing events related to cardiac development. **a** Volcano plots of the differential alternative splicing events identified in E13.5 *Nfatc1-Cre*;*Ptbp1*^*fl/fl*^ hearts and *Cdh5-CreERT2*;*Ptbp1*^*fl/fl*^ hearts compared with *Ptbp1*^*fl*/*fl*^ hearts, respectively. Significant alternative splicing events (FDR < 0.05 and | ΔPSI | ≥ 0.2) are colored as blue and red dots if *Ptbp1* deletion results in skipping or inclusion, respectively. *n* = 2–4 mice per group. PSI percent spliced in. **b** Violin plot of the distributions of differential alternative splicing event types in *Nfatc1-Cre*;*Ptbp1*^*fl*/*fl*^ and *Cdh5-CreERT2*;*Ptbp1*^*fl*/*fl*^ hearts, respectively. A3SS alternative 3’ splice site, A5SS alternative 5’ splice site, MXE mutually exclusive exon, RI retention intron, SE skipped exon. **c** Stacked bar graph showing the proportions of different types of differential alternative splicing events in *Nfatc1-Cre*;*Ptbp1*^*fl*/*fl*^ and *Cdh5-CreERT2*;*Ptbp1*^*fl*/*fl*^ hearts, respectively. **d** Gene Ontology (GO) enrichment analysis of genes with differential skipping exon (SE) events in *Nfatc1-Cre*;*Ptbp1*^*fl*/*fl*^ and *Cdh5-CreERT2*;*Ptbp1*^*fl*/*fl*^ hearts, respectively. GO analysis were performed using R package clusterProfiler. **e** Venn diagram showing the common significant SE events between *Nfatc1-Cre*;*Ptbp1*^*fl*/*fl*^ and *Cdh5-CreERT2*;*Ptbp1*^*fl*/*fl*^ hearts. Exons are required to change in the same direction to appear at the intersection of the two sets. **f** Circular plot showing the selected functional categories of common genes with differential SE events in E13.5 *Nfatc1-Cre*;*Ptbp1*^*fl/fl*^ and *Cdh5-CreERT2*;*Ptbp1*^*fl/fl*^ hearts. Source data are provided as a Source Data file.

To identify the mechanisms underlying abnormal ventricular development in *Ptbp1*-deficient mice, we performed RNA-seq and alternative splicing analyses of E13.5 hearts from *Nfatc1*-*Cre*;*Ptbp1*^*fl/fl*^, *Cdh5*-*CreERT2*;*Ptbp1*^*fl*/*fl*^ mice and *Ptbp1*^*fl*/*fl*^ mice. We identified 508 differential splicing events in *Nfatc1*-*Cre*;*Ptbp1*^*fl/fl*^ hearts and 769 differential splicing events in *Cdh5*-*CreERT2*;*Ptbp1*^*fl*/*fl*^ hearts (FDR value <0.05, |ΔPSI|value ≥0.2) (Fig. 6a). Classification of the alternative splicing events showed that the majority of them were cassette skipped exon (SE) events (Fig. 6b, c). We found that the genes with SE events were involved in biological processes associated with cardiac development, including cell morphogenesis, cell migration, cardiac muscle cell differentiation and cardiac muscle contraction (Fig. 6d).

**Fig. 7:**
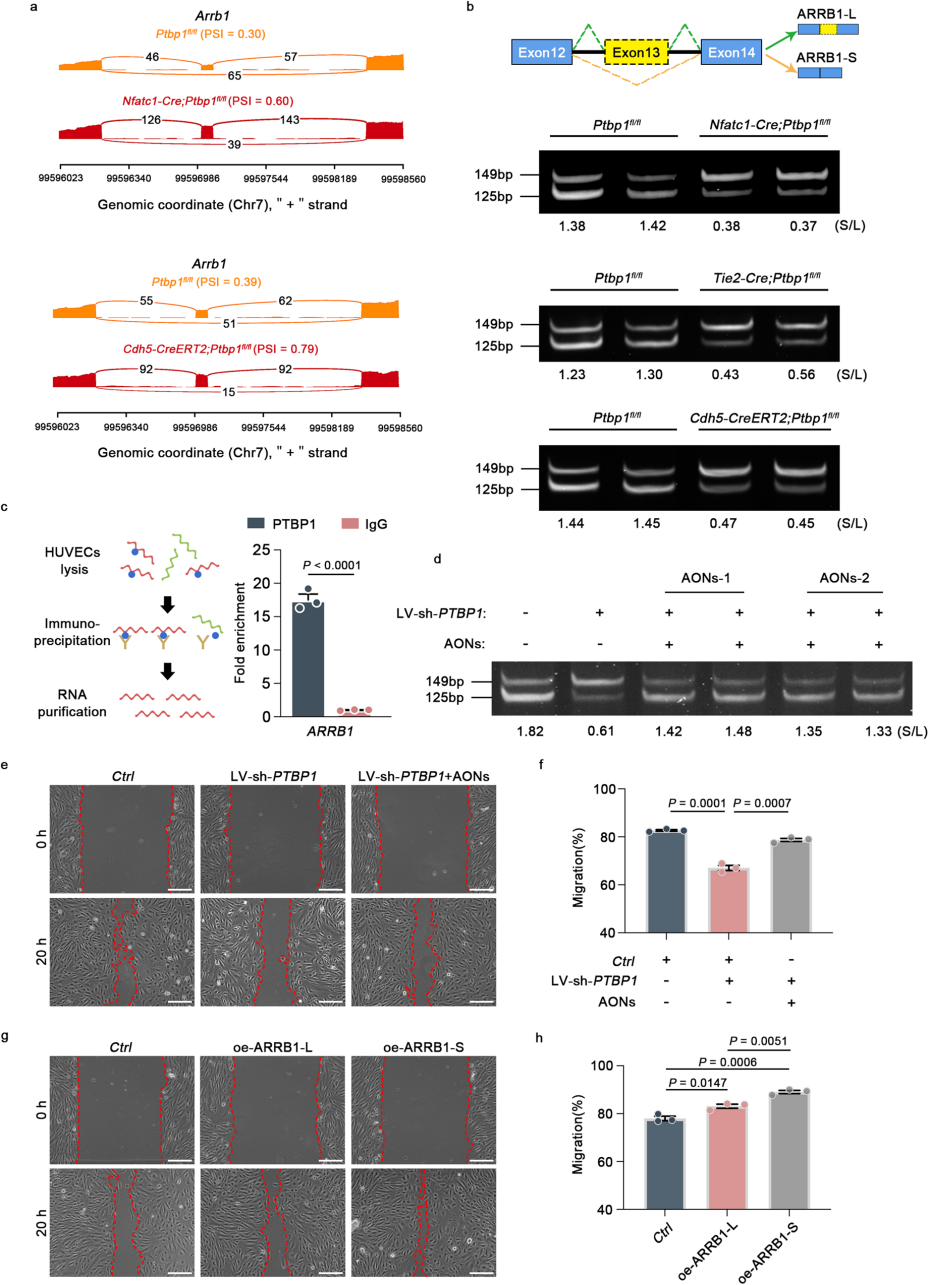
PTBP1 regulates the alternative splicing of *Arrb1* to control endothelial cell migration. **a** Representative images of the Sashimi plots showing the exon splicing of *Arrb1*. The Sashimi plots show the densities of exon-including and exon-skipping reads as determined by rMATS-turbo analysis. **b** Gel electrophoresis analysis of the ARRB1 isoforms in endothelial cells from *Nfatc1-Cre*;*Ptbp1*^*fl*/*fl*^*, Tie2-Cre*;*Ptbp1*^*fl/fl*^ and *Cdh5-CreERT2*;*Ptbp1*^*fl*/*fl*^ hearts. The values indicate expression ratios of the short ARRB1 isoform (ARRB1-S, lacking exon 13) and the long ARRB1 isoform (ARRB1-L, including exon 13) (S/L). **c** qRT-PCR analysis of *ARRB1* from RNA immunoprecipitation assay of HUVECs using anti-PTBP1. RNA enrichment is determined relative to the nontargeting IgG control. RIP assay was described in the schematic diagram. *n* = 3 independent experiments. **d** AONs transfection reverses the switching change of S/L ratio caused by *PTBP1* knockdown (LV-sh-*PTBP1*). The S/L values indicate ratios of ARRB1-S to ARRB1-L. AONs-1 and AONs-2 represent different oligonucleotide sequences **e**, **f** AONs rescued the decreased HUVEC migration caused by *PTBP1* knockdown (LV-sh-*PTBP1*). *n* = 3 independent experiments. Scale bars, 200 µm. LV-sh-*PTBP1*, lentivirus-shRNA-*PTBP1*; AONs, antisense oligonucleotides; *Ctrl*, control lentivirus-shRNA or control AONs. **g**, **h** ARRB1-L overexpression (oe-ARRB1-L) or ARRB1-S overexpression (oe-ARRB1-S) could promote the HUVEC migration. *n* = 3 independent experiments. Scale bars, 200 µm. *Ctrl* control lentivirus-vector. The data are presented as the mean ± s.e.m. *P* values were calculated by unpaired two-tailed *t* test. Source data are provided as a Source Data file.

Considering the similarity of the cardiac phenotypes of the two mouse strains, we overlapped the SEs from *Nfatc1*-*Cre*;*Ptbp1*^*fl/fl*^ mice with those of *Cdh5*-*CreERT2*;*Ptbp1*^*fl*/*fl*^ mice, revealing 18 common events (Fig. 6e). We found that the genes associated with these common events were related to cell migration, cell adhesion, protein metabolic processes and ionic channels (Fig. 6f). Moreover, the change of *Arrb1* alternative splicing was identified as the significantly affected common event (Fig. 7a). Further studies revealed that *Ptbp1* deletion decreased the expression ratio of the short ARRB1 isoform (ARRB1-S, lacking exon 13) and the long ARRB1 isoform (ARRB1-L, including exon 13) in the cardiac endothelial cells of *Nfatc1*-*Cre*;*Ptbp1*^*fl/fl*^ and *Cdh5*-*CreERT2*;*Ptbp1*^*fl*/*fl*^ mice, exhibiting a switching change (Fig. 7b). In addition, the alternation between the two ARRB1 isoforms was confirmed in the cardiac endothelial cells of *Tie2*-*Cre*;*Ptbp1*^*fl*/*fl*^ mice (Fig. 7b).

To further investigate the direct effects of PTBP1 on cardiac endothelial cells, we isolated endothelial cells from *Nfatc1-Cre*;*Ptbp1*^*fl/fl*^, *Cdh5-CreERT2*;*Ptbp1*^*fl/fl*^ and *Ptbp1*^*fl/fl*^ hearts and performed RNA-seq analysis. 673 differential splicing events in *Nfatc1-Cre*;*Ptbp1*^*fl/fl*^ mice and 457 differential splicing events in *Cdh5-CreERT2*;*Ptbp1*^*fl/fl*^ mice were identified, respectively (Supplementary Fig. 11a). The majority of differential splicing events were also classified as cassette SE events (Supplementary Fig. 11b, c). The genes with these SE events were involved in biological processes tightly associated with cardiac development (Supplementary Fig. 11d). Furthermore, we overlapped the SE events from the two endothelial-specific knockout mouse lines, which revealed 17 common events (Supplementary Fig. 11e). Importantly, the switching change between the two ARRB1 isoforms was also identified (Supplementary Fig. 11f), consistent with the alternative splicing analysis using heart tissue samples. In addition, we performed RIP assays using primary human umbilical endothelial cells (HUVECs), which revealed the direct binding of PTBP1 and *ARRB1* (Fig. 7c).

ARRB1 is an adapter protein best known for regulating GPCR signaling^28^. However, the alternative splicing of *Arrb1* in the heart has not been documented. To determine whether the change of ARRB1-S to ARRB1-L (S/L) ratio affected the migration of endothelial cells, we designed antisense oligonucleotides (AONs), the specific nucleic acid fragments which suppressed ARRB1-L expression by binding to its complementary sequences in exon 13. PCR analysis showed that the switching change in the ratio of S/L caused by *PTBP1* knockdown was significantly reversed after the transfection of AONs in HUVECs (Fig. 7d). Importantly, the scratch assays showed that the reduced cell migration caused by *PTBP1* knockdown in HUVECs was significantly rescued after AONs transfection (Fig. 7e, f), suggesting that the ratio of S/L is critical to the regulation of *PTBP1* in endothelial cell migration.

In addition, we also investigated the effect of ARRB1-L or ARRB1-S on HUVEC migration, respectively. The results showed that overexpression of ARRB1-L or ARRB1-S promoted the migration of HUVECs, while overexpression of ARRB1-S exhibited a more significant effect than that of ARRB1-L (Fig. 7g, h and Supplementary Fig. 12). Above all, these data revealed that PTBP1 regulated the alternative splicing of *Arrb1*, thereby controlling the migration of endothelial cells.

### The alteration of gene expression caused by *Ptbp1* deletion

Alternative splicing plays a pivotal role in cell fate determination and organ morphogenesis by controlling enormously diverse transcriptomes^17,29–31^. To further explore the contribution of *Ptbp1* deletion to the occurrence of LVNC, we further analyzed the differentially expressed genes (DEGs) in cardiac endothelial cells of *Nfatc1*-*Cre*;*Ptbp1*^*fl/fl*^, *Cdh5*-*CreERT2*;*Ptbp1*^*fl*/*fl*^ and *Ptbp1*^*fl*/*fl*^ mice at E13.5. As shown in Supplementary Figs. 13 and  14, 310 DEGs in *Nfatc1*-*Cre*;*Ptbp1*^*fl/fl*^ mice and 396 DEGs in *Cdh5*-*CreERT2*;*Ptbp1*^*fl*/*fl*^ hearts were identified, respectively.

GO analysis revealed that the DEGs in *Nfatc1*-*Cre*;*Ptbp1*^*fl/fl*^ mice were tightly associated with cell migration, cell differentiation, animal organ morphogenesis and embryonic organ development (Supplementary Fig. 13b). For the *Cdh5*-*CreERT2*;*Ptbp1*^*fl*/*fl*^ hearts, the DEGs were mainly involved in biological processes including signal transduction, multicellular organism development, cell migration and muscle organ morphogenesis (Supplementary Fig. 14b). Moreover, Kyoto Encyclopedia of Genes and Genomes (KEGG) analysis revealed the involvement of the DEGs in heart development-related pathways such as Hippo signaling pathways (Supplementary Figs. 13c and 14c)^32^. It has been reported that transcription factors such as *Hey2*^16^, *Tbx2*0^27^, *N-Myc*^16^, *Nkx2.5*^33^, *Casz1*^34^, *Jarid2*^34^, and *Prdm16*^25^ are tightly related to the pathogenesis of LVNC. We then examined their expression in the hearts of three endothelial-specific *Ptbp1* knockout mice. Results showed that the expression of *N-Myc* and *Prdm16* in *Nfatc1-Cre*;*Ptbp1*^*fl/fl*^ hearts and the expression of *Jarid2* in *Cdh5-CreERT2*;*Ptbp1*^*fl/fl*^ hearts were significantly decreased (Supplementary Fig. 15). Together, these results showed that the alternation of gene expression induced by *Ptbp1* deletion was tightly related to the pathogenesis of LVNC.

## Discussion

We demonstrated an uncovered role of the alternative splicing factor PTBP1 in ventricular chamber development. First, PTBP1 was shown to colocalize with endothelial cells during heart development. Endothelial-specific knockout of *Ptbp1* resulted in the LVNC phenotype. Second, *Ptbp1* deficiency affected the migration of endothelial cells, thereby disrupting the proliferation of cardiomyocytes. Third, PTBP1 regulated the expression ratios between the two ARRB1 isoforms to control endothelial cell migration. Finally, a series of alternative splicing events and DEGs induced by *Ptbp1* deletion were shown to be involved in LVNC-related biological processes.

This study revealed the characteristic localization of PTBP1 in the embryonic heart. As an alternative splicing factor, PTBP1 is ubiquitously expressed in various tissues and organs^20,35–37^. We found that PTBP1 primarily colocalized with endothelial cells during cardiac development but was nearly undetectable in cardiomyocytes. Due to the characteristics of endothelial cells, especially their migration property during cardiac development, the distribution of endothelial cells is unique and dynamic at different embryonic stages. At the early developmental stage, endothelial cells are shown to be enriched in the endocardium and endocardial cushions. As development proceeds, endothelial cells gradually migrate outward from the endocardium to the compact myocardium^10^. Deciphering the colocalization of PTBP1 with endothelial cells in different embryonic periods might provide important insights into the biological processes related to cardiac development.

Researchers have focused on the important roles of endothelial cells, one of which is to regulate cardiomyocyte proliferation during heart development^14,24,38^. Here, we found that *Ptbp1* deficiency reduced the migration of endothelial cells and affected cardiomyocyte proliferation. Differential changes in the proliferation of cardiomyocytes in compact and trabecular myocardium are one of the manifestations of LVNC^25–27^. A significant abnormality in cardiomyocyte proliferation was observed in endothelial-specific *Ptbp1* knockout mice, revealing that *Ptbp1* deficiency in endothelial cells led to ventricular noncompaction. Endothelial cells are emerging as important signaling sources that coordinate cardiac cell populations^11,12,39^. In our studies, we identified a critical function of PTBP1 in endothelial cell-mediated cardiomyocyte proliferation. The role of PTBP1 in the interactions between endothelial cells and other cardiac cells warrants further exploration. Notably, we focused on the migration of cardiac endothelial cells to explore the mechanism underlying the abnormalities of ventricular chamber development. However, the abnormal cardiac endothelial migration may not fully account for the increased embryonic lethality in *Tie2*-*Cre*;*Ptbp1*^*fl*/*fl*^ and *Cdh5*-*CreERT2*;*Ptbp1*^*fl*/*fl*^ mice. Considering that *Cdh5-CreERT2* and *Tie2-Cre* mediate the gene knockout in pan-endothelial cells, not just in the cardiac endothelial cells, we suspect that the systemic vascular defects are one of the important causes of embryonic death in *Tie2*-*Cre*;*Ptbp1*^*fl*/*fl*^ and *Cdh5*-*CreERT2*;*Ptbp1*^*fl*/*fl*^ mice.

Although alternative splicing events have been gradually identified in developing organs, including the heart^30,31^, the functional consequences of alternative splicing are largely unclear. The alternative splicing analysis of endothelial-specific *Ptbp1*-deficient hearts revealed a set of splicing events that are involved in heart development-related cellular processes, signaling and diseases. Furthermore, the switching change of the expression ratio between the two ARRB1 isoforms was identified in *Ptbp1*-deficient cardiac endothelial cells and was critical to the regulation of endothelial cell migration mediated by PTBP1. As an adapter protein, ARRB1 is best known as a regulator of GPCR signaling and has been found to be involved in signaling pathways related to vascular function^40^, while our study uncovered the role of ARRB1 isoforms in cardiac development. The splicing event changes identified in our work indicate the important transcript networks underlying cardiac development.

The functional properties of ARRB1 alternative splicing in diseases remain unknown. It has been identified that the functional domains of ARRB1 can bind to key molecules in multiple signaling pathways, suggesting their potential role in related pathological processe^41–44^. We speculate that *Ptbp1* deficiency leads to the alternative splicing changes in *Arrb1*, which alters the expression of the Arrestin_C domain located on the ARRB1-L isoform, affects the binding of ARRB1 to downstream molecules associated with endothelial cell function and is thereby involved in the regulation of heart development. Moreover, the other common genes with differential SE events in *Nfatc1-Cre*;*Ptbp1*^*fl/fl*^ and *Cdh5-CreERT2*;*Ptbp1*^*fl/fl*^ hearts were involved in embryonic development, cell migration, cell differentiation, cell growth and proliferation. However, the role of these genes in cardiac development has not been fully studied. The functional differences of these genes and their isoforms may be responsible for the phenotypic differences between distinct mouse strains, which need to be further explored.

Clinically, a minority of LVNC patients have biventricular noncompaction, while most patients have a phenotype only in the left ventricle. Here, we examined the phenotypes both in the left and right ventricles of *Ptbp1* deficiency mice, which revealed their biventricular noncompaction. In addition, there are many genes, such as *Mib1*, *Nb/Nbl*, and *Nae1*, which have been recognized as the key regulators in the pathogenesis of LVNC^16,45,46^. The knockdown of these genes in mice all manifests biventricular noncompaction. These data suggest that the same molecular defect can cause large phenotypic differences between different species. The phenotype inconsistency of LVNC between the animal model and human disease may be one of the main limitations for its pathogenesis exploration.

In summary, we demonstrated the important role of PTBP1 as a splicing factor in the pathogenesis of LVNC. The specific knockout of *Ptbp1* in endothelial cells resulted in the disruption of endothelial cell migration and cardiomyocyte proliferation, thereby affecting ventricular chamber development. We also observed that *Ptbp1* deficiency affected multiple signaling pathways involved in other cardiac pathological processes, providing insight into PTBP1 and heart diseases. In addition, our data suggest that defects in the spatiotemporal interactions of different cell types in the embryonic stage are potentially important mechanisms underlying organ development disorders.

## Methods

### Animals

All procedures involving animals conformed to the Guidelines for the Care and Use of Laboratory Animals established by the U.S. National Institutes of Health (National Academies Press; 2011) and were approved by Tongji University School of Medicine. The mice were bred on a 12/12 h light/dark cycle at the temperature of 18–23 °C and a humidity of 40–60%. The mice in our study were age-matched, and the genotypes were blinded to the researchers.

### Mouse alleles and transgenic lines

All mice were maintained on a mixed C57BL6 genetic background. The mice were genotyped by PCR using genomic DNA isolated from their tails. For the construction of *Ptbp1*-floxed mice, the target gene *Ptbp1* was modified with flox using CRISPR/Cas9 technique. The construction process is briefly described as follows: the gRNA was transcribed in vitro to construct a donor vector. Cas9, gRNA, and donor vector were then simultaneously injected into mouse-fertilized eggs. Under the guidance of gRNA, Cas9 protein binds to the target site and then causes DNA double-strand break. The donor vector can repair the broken double-strand by homologous recombination, leading to flox modification of the target gene. The *Ptbp1*-floxed mouse line was constructed in GemPharmatech Co., Ltd.

*Ptbp1*-floxed mice were genotyped with the following primers: forward: 5’-CAGTAAACCAAGCAGAGGGTTACAC-3’ and reverse: 5’-ACTGCTGATGGCAGGCAAAG-3’. The WT allele was determined to be 253 bp, and the mutant allele was determined to be 346 bp. Endothelial-specific *Ptbp1*-deficient mice were generated by crossing *Ptbp1*^*fl/fl*^ mice with *Tie2-Cre* (Jackson Laboratories Stock number 008863, B6.Cg-Tg (Tek-cre)1Ywa/J) or *Cdh5-CreERT2* mice^47^, respectively. *Tie2-Cre* and *Cdh5-CreERT2* mice were genotyped with the following primers: *Cdh5-CreERT2* (forward: 5’-TCCTGATGGTGCCTATCCTC-3’ and reverse: 5’-CCTGTTTTGCACGTTCACCG-3’); *Tie2-Cre* (forward: 5’-CGGGAAGTCGCAAAGTTGTG-3’ and reverse: 5’-CGCATAACCAGTGAAACAGCATTG-3’). For *Cdh5-CreERT2* mice, the mutant allele was determined to be 548 bp. For *Tie2-Cre* mice, the mutant allele was determined to be 450 bp. An endocardial-specific *Ptbp1*-deficient mouse line was generated by crossing *Ptbp1*^*fl/fl*^ mice with *Nfatc1-Cre* mice. The *Nfatc1-Cre* mouse line was kindly provided by Bin Zhou, the Institute for Shanghai Institute of Biochemistry and Cell Biology. *Nfatc1-Cre* mice were genotyped with the following primers: 5′-CCACCCCCTCAAAGAAAAGC-3′, 5′-CCTCACATTGCCAAAAGACGG-3′, and 5′-CAGGATAACAACCGACTCTGCTCTC-3′. The WT allele was identified as 581 bp, and the mutant allele was determined to be 472 bp.

To trace endothelial lineages, *Tie2-Cre, Cdh5-CreERT2,* and *Nfatc1-Cre* mice were crossed with *Rosa26*-tdTomato mice. The *Rosa26*-tdTomato mouse line was kindly provided by Bin Zhou, the Institute for Shanghai Institute of Biochemistry and Cell Biology. The *Rosa26*-tdTomato mice were genotyped with the following primers: 5’-AAGGGAGCTGCAGTGGAGTA-3’, 5’-CCGAAAATCTGTGGGAAGTC-3’, 5’-GGCATTAAAGCAGCGTATCC-3’ and 5’-CTGTTCCTGTACGGCATGG-3’. The WT allele was determined to be 297 bp, and the mutant allele was determined to be 196 bp. In this mouse line, the endothelium was identified by the RFP protein under tamoxifen recombination.

To achieve the recombination of floxed alleles in pregnant mice, tamoxifen (Sigma, T5648) (dissolved in 90% peanut oil and 10% alcohol) was administered by oral gavage at a dose of 0.05 mg/g at 8.5 days of gestation.

### Histology

Embryos or embryonic hearts were harvested at the indicated times of gestation (morning of plug designated as E0.5) and fixed in 4% paraformaldehyde (PFA, Sigma, 158127) overnight at 4 °C. The next day, the samples were washed with PBS, dehydrated in ascending concentrations of ethanol, treated with xylene, embedded in paraffin, and sectioned at a thickness of 5 µm. The sections were stained with hematoxylin and eosin (H&E) according to previously published methods^48^ and visualized using a microscope for routine histological analysis.

### Immunofluorescence

Paraffin-embedded embryos and heart slides were deparaffinized, rehydrated, and incubated in a heated water bath for antigen retrieval in citrate solution before being washed in 0.1% PBST, and then incubated with 10% fetal bovine serum for 1 h at room temperature. Then, the slides were incubated with primary antibodies overnight at 4 °C. The following day, the slides were washed with 0.1% PBST three times and incubated with the corresponding secondary antibodies for 1 h at room temperature. For the detection of apoptosis, the slides were incubated with In Situ Cell Death Detection Kit, TMR red (Roche, 12156792910) for 30 min according to the manufacturer’s instructions. After three washes in 0.1% PBST, nuclei were stained with DAPI (Sigma, D9542). Immunofluorescence images were acquired with either a fluorescence microscope (Leica, DMi8) or a confocal microscope (Leica, TCS SP8).

The following primary antibodies were used: cTnT (Abcam, ab8295, 1:50), PTBP1 (Abcam, ab133734, 1:100), RFP (Abcam, ab124754, 1:200), PTBP1 (Sigma, WH0005725M1, 1:150), EMCN (Santa Cruz, sc-65495, 1:200), CX40 (Thermo, 37-8900), pH3 (Abcam, ab32107, 1:1000), NKX2.5 (Abcam, ab97355, 1:100), VE-Cadherin (R&D Systems, AF1002-SP, 1:100), ERG (Abcam, ab92513, 1:200), pH3 (CST, 9706S, 1:100).

The secondary antibodies used in the experiment included Goat anti-Mouse IgG Alexa Fluor® 488 (Abcam, ab150113, 1:200), Goat anti-Rabbit IgG Alexa Fluor® 555 (Abcam, ab150078, 1:200), Goat anti-Mouse IgG Alexa Fluor® 555 (Abcam, ab150118, 1:200), Goat anti-Rabbit IgG Alexa Fluor® 488 (Abcam, ab150081, 1:200), Goat Anti-Rat IgG H&L (Alexa Fluor® 488) preadsorbed (Abcam, ab150165, 1:200) and Donkey Anti-Goat IgG Alexa Fluor® 555 (Abcam, ab150134, 1:200).

### Quantification of the thickness of compact myocardium and trabecular myocardium

To visualize the structure of embryonic ventricles, immunostaining of the paraffin sections was performed as previously described^24^. The antibody EMCN was used to label endocardial cells, and DAPI was used for nuclear labeling. The thickness of the compact myocardium (CM) and trabecular myocardium (TM) in tissue sections from equivalent coronal planes of the heart were then measured by ImageJ software. For each parameter, six heart sections per mouse was measured along the lateral sides of the heart and averaged individually.

### Quantification of endocardial complexity

The endocardial complexity was quantified as previously described^49^. To measure endocardial branch points, the total area covered by endocardial network and total length of the endocardial network, the transverse heart sections of E13.5 control and *Ptbp1* knockout embryonic hearts were stained with anti-endomucin for visualization of endocardial networks. Images were used for measurements with AngioTool.

### Whole-mount immunofluorescence staining

Whole-mount RFP staining was performed as previously described^24^. Briefly, embryonic hearts were collected and fixed in 4% PFA overnight at 4 °C. The next day, the samples were washed with PBS three times, followed by serial methanol dehydration. Then, the samples were bleached in 5% hydrogen peroxide and 100% methanol for 2 h at 4 °C, followed by rehydration. Then, the samples were blocked in PBS containing 10% FBS and 0.1% Triton X-100 for 1 h at 4 °C. the samples were then incubated in block solution containing RFP antibody (Abcam, ab124754, 1:200) overnight at 4 °C, followed by five times washes with PBS containing 0.1% Triton X-100. Then, the samples were incubated with secondary antibody in block solution for 2 h, followed by five times washes with PBS containing 0.1% Triton X-100. Images were taken under confocal microscope (Leica, TCS SP8).

Endothelial cell coverage was calculated as the total area of heart ventricles occupied by endothelial cells. ImageJ software was used to circumscribe the whole heart and areas containing endothelial cells.

### Migration assays

Human umbilical vein cells (HUVECs, PromoCell, C-12208) were cultured in endothelial cell growth medium (PromoCell, C-22010). To knockdown the expression of PTBP1, HUVECs were infected with lentivirus-shRNA-*PTBP1* (LV-sh-*PTBP1*) (Gene Pharma, Inc.) at a multiplicity of infection (MOI) of 50 at 37 °C for 24 h, and then cultured with fresh medium for another 48 h at 37 °C in a 5% CO2 incubator before subsequent experiments. The cells infected with control lentivirus-shRNA (Gene Pharma, Inc.; MOI, 50) were used as a negative control. For the migration assay, HUVECs were incubated with 10 μg/mL mitomycin C (MedChemExpress) at 37 °C in a 5% CO2 atmosphere for 2 h^50^. The LV-sh-*PTBP1* sequences were as follows: 5’-CGGCACAGTGTTGAAGATCAT-3’. For the overexpression of ARRB1-L or ARRB1-S, HUVECs were infected with ARRB1-L overexpression lentivirus or ARRB1-S overexpression lentivirus. At 72 h after infection, the growth area was scratched. The cells were then photographed in five different fields at 0 h and 20 h after scratching using a Leica DMi8 microscope, respectively. Cell migration ability was calculated as a percentage of the wound area difference between 0 h and 20 h to the wound area at 0 h by ImageJ software.

### Explant culture, immunostaining, and quantification

The left ventricles without atria were isolated from E11.5 embryos, rinsed with PBS to remove blood cells and placed in the Matrigel (Corning, 356237) with culture media (EGM-2 MV, Clonetics, CC-4147) in 24-well plates (Costar, 3524). Explants were cultured in 5% CO_2_ at 37 °C for 2 or 5 days before samples were fixed with 4% PFA for 15 min. After washing the explants with 0.5% PBST three times, immunofluorescence staining was performed directly within the 24-well culture plates. Explants were incubated with primary antibodies in 0.5% PBST overnight at 4 °C. After that, explants were washed with 0.5% PBST six times for 6 h and then incubated with corresponding secondary antibodies in 0.5% PBST overnight at 4 °C. The next day, explants were washed three times and stained with DAPI to label nuclei. After three washes with PBS for 15 min, the explants were placed in PBS and photographed using a confocal microscope (Leica, TCS SP8). The primary antibodies included ERG (Abcam, ab92513, 1:200) and cTnT (Abcam, ab8295, 1:50). The secondary antibodies used in the experiment included Goat anti-Mouse IgG Alexa Fluor® 555 (Abcam, ab150118, 1:200) and goat anti-rabbit IgG Alexa Fluor® 488 (Abcam, ab150081, 1:200).

To measure the distance of endothelial cells migration from cultured ventricular tissues, explants were stained with ERG. The distance of endothelial cells migration from the inside line of ERG-positive cells in the explants to the maximum distance reached by the endothelial cells was measured in three fields per sample^24^. The distance of endothelial cell migration was measured by ImageJ software.

### EdU assay

For explant cultures, the medium was replaced with 0.5 ml of medium containing 200 ng of EdU 30 min before fixation. For the detection of EdU incorporation, explant slides were incubated with Click-iT EdU 555 Imaging Kit reagents (Thermo Fisher Scientific, c10338) for 30 min according to the manufacturer’s instructions.

### RNA sequencing and data analysis

E13.5 embryos were collected from pregnant *Nfatc1-Cre;Ptbp1*^*fl/fl*^, *Cdh5-CreERT2;Ptbp1*^*fl/fl*^ and *Ptbp1*^*fl/fl*^ mice. Total RNA from whole hearts or isolated cardiac endothelial cells was extracted using the Trizol reagent (Thermo Fisher Scientific), and the RNA integrity was evaluated using an Agilent 2100 Bioanalyzer (Agilent Technologies, Santa Clara, CA, USA). The samples with an RNA integrity number (RIN) ≥ 7 were subjected to subsequent analysis. The libraries were constructed using the TruSeq Stranded mRNA LTSample Prep Kit (Illumina, San Diego, CA, USA) according to the manufacturer’s instructions. Then, these libraries were sequenced on the Illumina sequencing platform (Illumina HiSeq X Ten), and 150 bp paired-end reads were generated. The clean reads were mapped to the GRCm38.p6 genome using HISAT2^51^. The FPKM value of each gene was calculated using Cufflinks, and the read counts for each gene were obtained by HTSeqcount.

Alternative splicing analysis was conducted using rMATS (version 4.1.0)^52^ with the following nondefault parameters: read Length 150, cstat 0.001, libType fr-firststrand. The FDR and changes in the exon inclusion level (ΔPSI) were calculated for each event in each condition. On the basis of the FDR value (<0.05) and the | ΔPSI | value (≥0.2), we identified significant differential splicing events. Differential expression analysis was performed using the DESeq (2012) R package. |log_2_ FC| > 0.58 and *P* < 0.05 were set as the threshold for significantly differential expression. Hierarchical cluster analysis of differentially expressed genes (DEGs) was performed to demonstrate the expression pattern of genes in different groups. GO enrichment and KEGG pathway enrichment analysis of DEGs were performed, respectively, using R based on the hypergeometric distribution.

### Assay of splicing with semi-quantitative RT-PCR

For alternative splicing analysis, total RNA was extracted from mouse cardiac endothelial cells and HUVECs using RNAiso Reagent (Takara, 9112). cDNA synthesis was performed using PrimeScript™ RT Master Mix (Takara, RR036A). PCR amplification was performed using Phanta Max Super-Fidelity DNA Polymerase (Vazyme, P505-d1), and the samples were run on a ProFlex™ PCR system (Thermo Fisher). The primers were specifically designed to amplify the alternatively spliced exon of *Arrb1* (forward: 5′-GGGCATCATCGTTTCCTACA-3′ and reverse: 5′-CTTCCCGATGTGGGGGCTCC-3′); *ARRB1* (forward: 5′-GGGGATCATTGTTTCCTACA-3′ and reverse: 5′-CTTCCCGATGCGGGGGTTCC-3′). The PCR product was then visualized on a 12% PAGE gel using the ChemiDoc™ Imaging System (Bio-Rad) and quantified according to ImageJ software.

### Isolation of cardiac endothelial cells

Hearts from E13.5 embryos were collected, cut into pieces, and incubated in 0.4 ml of dissociation solution (collagenase type II, Worthington, LS004176, 1 mg/ml; DNase, Sigma, D8764, 10 µg/ml) at 37 °C for 45 min. Once digested, 0.2 ml of fetal bovine serum (Excell, FCS500) was added to terminate the digestion, and the digested heart tissues were filtered through a 40-µm cell strainer. After centrifugation, the supernatant was removed, and the cells were resuspended in PEB buffer consisting of PBS, 1 mM EDTA (Dingguo, NEP046), and 1% bovine serum albumin (BSA, Beyotime, ST023). Ten microliters of CD31 antibody-coupled magnetic beads (Invitrogen, 11035) were added to the suspension and incubated for 1 h at room temperature. Bead-bound endothelial cells were isolated magnetically and washed 3 times with PEB buffer. Endothelial cells were then directly collected in 1 ml of RNAiso Reagent (Takara, 9109) for RNA extraction.

### RNA immunoprecipitation assay

RIP was performed using a Magna RIP RNA-Binding Protein Immunoprecipitation Kit (Millipore, 17-700) according to the manufacturer’s instructions. HUVECs in 15-cm dishes were collected and resuspended in cold RIP lysis buffer, and then stored at –80 °C overnight. Magnetic beads were preincubated with 1 µg of PTBP1 antibody (Invitrogen, 32-4800) or with 1 µg of control IgG (Millipore, 03-241). Next, the frozen homogenates were thawed quickly and centrifuged at 18,000 × *g* for 10 min, and 10 µl of the supernatant was stored as input. Then, for each RIP reaction, 100 µl of supernatant was incubated overnight with the magnetic bead-antibody complex at 4 °C. On the second day, the RNA/protein immunocomplex was extensively washed with RIP Wash Buffer (provided in the kit). The cross-linking was reversed by incubation with proteinase K. The immunoprecipitated RNA was purified through phenol:chloroform:isoamyl alcohol (125:24:1) isolation. The purified immunoprecipitated RNA was reverse-transcribed into cDNA using an RNA-to-cDNA Kit (Takara, RR036A). qRT-PCR was performed using this cDNA as a template to quantify the *ARRB1* mRNA; the following primers were used: *ARRB1* (forward: 5’-TACAGTCGTTCCCACCGG-3’ and reverse: 5’-GACGCACAGAATTCCGCT-3’) and *GAPDH* (forward: 5’-GTCTCCTCTGACTTCAACAGCG-3’ and reverse: 5’- ACCACCCTGTTGCTGTAGCCAA-3’).

### qRT-PCR

Total RNA was extracted from cardiac endothelial cells and heart tissues using RNAiso Reagent (Takara, 9109), and cDNA was generated using PrimeScript^TM^ cDNA RT Master Mix (Takara, RR036A). qRT-PCR was performed on a StepOnePlus Real-Time PCR system (Applied Biosystems) with SYBR Green Supermix (Applied Biosystems, A25742). All procedures were performed according to the manufacturers’ manuals. The PCR primers are listed in Supplementary Table 4.

### Western blot analysis

Proteins were extracted on ice using a RIPA lysis buffer (Beyotime, P0013C) containing protease inhibitors (Roche, 04693132001). Equal amounts of total proteins (60 µg) were mixed and dissolved in 4× SDS/PAGE sample buffer and heated to 100 °C for 5 min. The proteins were then separated on NuPAGE 10% Bis-Tris Gels (Invitrogen, NP0315BOX) and electrophoretically transferred onto polyvinylidene fluoride membranes. The membranes were immunoblotted overnight at 4 °C with the following antibodies: ARRB1 (Rabbit, Abcam, ab32099, 1:500), GAPDH (Mouse, Proteintech, 60004-1-Ig, 1:10,000). The following day, after washing three times with TBST, the membranes were incubated with conjugated fluorescent secondary antibody (Invitrogen), and the blots were imaged using a ChemiDoc™ Imaging Systems (Bio-Rad). The secondary antibodies used in the experiment included Goat anti-Mouse IgG (H + L) Highly Cross-Adsorbed Secondary Antibody, Alexa Fluor Plus 800 (Invitrogen, A32730, 1:10,000) and Goat anti-Rabbit IgG (H + L) Highly Cross-Adsorbed Secondary Antibody, Alexa Fluor Plus 800 (Invitrogen, A32735, 1:10,000).

### Statistics and reproducibility

Statistical analysis was performed using Prism 8.0 (GraphPad Software). All data are presented as the mean  ± s.e.m. Unpaired two-tailed *t* tests and *Chi*-square tests were used to assess statistical significance. *P* < 0.05 was considered statistically significant. The representative data shown as microscopy images, gel electrophoresis images, and H&E staining images were obtained from at least three mice per group or at least three independent experiments (for Figs. 1a–b, 2b, e, 3a, c, 5d, 7b, d and Supplementary Figs. 1a–d, 2a–d, 4a–b).

### Reporting summary

Further information on research design is available in the Nature Portfolio Reporting Summary linked to this article.

## Supplementary information

Supplementary Information

Reporting Summary

## Data Availability

The authors declare that the data supporting the findings of this study are available within the article and Supplementary Information files. The RNA-seq data have been deposited in the NCBI Sequence Read Archive under accession numbers PRJNA772292 and PRJNA862199. The accessible link for GRCm38.p6 database used in this study is https://www.gencodegenes.org/mouse/release_M20.html. All remaining data will be available from the corresponding author upon reasonable request. Source data are provided with this paper.

## Source data

Source Data

## References

[1] Günthel M, Barnett P, Christoffels VM (2018). Development, proliferation, and growth of the mammalian heart. Mol. Ther..

[2] Sizarov A (2011). Formation of the building plan of the human heart: morphogenesis, growth, and differentiation. Circulation.

[3] Dong Y, Qian L, Liu J (2021). Molecular and cellular basis of embryonic cardiac chamber maturation. Semin. Cell Dev. Biol..

[4] Han P (2016). Coordinating cardiomyocyte interactions to direct ventricular chamber morphogenesis. Nature.

[5] Towbin JA, Lorts A, Jefferies JL (2015). Left ventricular non-compaction cardiomyopathy. Lancet.

[6] Finsterer J, Stöllberger C, Towbin JA (2017). Left ventricular noncompaction cardiomyopathy: cardiac, neuromuscular, and genetic factors. Nat. Rev. Cardiol..

[7] Hussein A, Karimianpour A, Collier P, Krasuski RA (2015). Isolated noncompaction of the left ventricle in adults. J. Am. Coll. Cardiol..

[8] Coris EE, Moran BK, De Cuba R, Farrar T, Curtis AB (2016). Left ventricular non-compaction in athletes: to play or not to play. Sports Med..

[9] Arbustini E, Favalli V, Narula N, Serio A, Grasso M (2016). Left ventricular noncompaction: a distinct genetic cardiomyopathy?. J. Am. Coll. Cardiol..

[10] Zhang H, Lui KO, Zhou B (2018). Endocardial cell plasticity in cardiac development, diseases and regeneration. Circ. Res..

[11] Tian Y, Morrisey EE (2012). Importance of myocyte-nonmyocyte interactions in cardiac development and disease. Circ. Res..

[12] Miao Y (2020). Intrinsic endocardial defects contribute to hypoplastic left heart syndrome. Cell Stem Cell.

[13] Luxán G, D’Amato G, MacGrogan D, de la Pompa JL (2016). Endocardial Notch signaling in cardiac development and disease. Circ. Res..

[14] D’Amato G (2016). Sequential Notch activation regulates ventricular chamber development. Nat. Cell Biol..

[15] Del Monte-Nieto (2018). Control of cardiac jelly dynamics by NOTCH1 and NRG1 defines the building plan for trabeculation. Nature.

[16] Luxán G (2013). Mutations in the NOTCH pathway regulator MIB1 cause left ventricular noncompaction cardiomyopathy. Nat. Med..

[17] Baralle FE, Giudice J (2017). Alternative splicing as a regulator of development and tissue identity. Nat. Rev. Mol. Cell Biol..

[18] Ule J, Blencowe BJ (2019). Alternative splicing regulatory networks: functions, mechanisms, and evolution. Mol. Cell.

[19] van den Hoogenhof MM, Pinto YM, Creemers EE (2016). RNA splicing: regulation and dysregulation in the heart. Circ. Res..

[20] Monzón-Casanova E (2018). The RNA-binding protein PTBP1 is necessary for B cell selection in germinal centers. Nat. Immunol..

[21] Zhang H (2017). Metabolic and proliferative state of vascular adventitial fibroblasts in pulmonary hypertension is regulated through a MicroRNA-124/PTBP1 (polypyrimidine tract binding protein 1)/pyruvate kinase muscle axis. Circulation.

[22] Shibayama M (2009). Polypyrimidine tract-binding protein is essential for early mouse development and embryonic stem cell proliferation. FEBS J..

[23] Dye B, Lincoln J (2020). The endocardium and heart valves. Cold Spring Harb. Perspect. Biol..

[24] Rhee S (2018). Endothelial deletion of Ino80 disrupts coronary angiogenesis and causes congenital heart disease. Nat. Commun..

[25] Wu T (2022). PRDM16 is a compact myocardium-enriched transcription factor required to maintain compact myocardial cardiomyocyte identity in left ventricle. Circulation.

[26] Gan P (2022). RBPMS is an RNA-binding protein that mediates cardiomyocyte binucleation and cardiovascular development. Dev. Cell.

[27] Kodo K (2016). iPSC-derived cardiomyocytes reveal abnormal TGF-β signalling in left ventricular non-compaction cardiomyopathy. Nat. Cell Biol..

[28] Shukla AK (2013). Structure of active β-arrestin-1 bound to a G-protein-coupled receptor phosphopeptide. Nature.

[29] Liu Z (2017). Single-cell transcriptomics reconstructs fate conversion from fibroblast to cardiomyocyte. Nature.

[30] Grifone R, Shao M, Saquet A, Shi DL (2020). RNA-binding protein Rbm24 as a multifaceted post-transcriptional regulator of embryonic lineage differentiation and cellular homeostasis. Cells.

[31] Cooper TA (2005). Alternative splicing regulation impacts heart development. Cell.

[32] Tian X (2017). Identification of a hybrid myocardial zone in the mammalian heart after birth. Nat. Commun..

[33] Choquet C (2018). Deletion of Nkx2-5 in trabecular myocardium reveals the developmental origins of pathological heterogeneity associated with ventricular non-compaction cardiomyopathy. PLoS Genet..

[34] Liu Z (2014). Essential role of the zinc finger transcription factor Casz1 for mammalian cardiac morphogenesis and development. J. Biol. Chem..

[35] Romanelli MG, Diani E, Lievens PM (2013). New insights into functional roles of the polypyrimidine tract-binding protein. Int. J. Mol. Sci..

[36] Caruso P (2017). Identification of MicroRNA-124 as a major regulator of enhanced endothelial cell glycolysis in pulmonary arterial hypertension via PTBP1 (polypyrimidine tract binding protein) and pyruvate kinase M2. Circulation.

[37] Georgilis A (2018). PTBP1-mediated alternative splicing regulates the inflammatory secretome and the pro-tumorigenic effects of senescent cells. Cancer Cell.

[38] Zhang Y (2010). Foxp1 coordinates cardiomyocyte proliferation through both cell-autonomous and nonautonomous mechanisms. Genes. Dev..

[39] de la Pompa JL, Epstein JA (2012). Coordinating tissue interactions: Notch signaling in cardiac development and disease. Dev. Cell.

[40] Ma Z (2019). Vascular endothelial growth factor receptor 3 regulates endothelial function through β-Arrestin 1. Circulation.

[41] Kallifatidis G, Mamouni K, Lokeshwar BL (2020). The role of β-arrestins in regulating stem cell phenotypes in normal and tumorigenic cells. Int. J. Mol. Sci..

[42] Feng Y (2007). The LIM protein, Limd1, regulates AP-1 activation through an interaction with Traf6 to influence osteoclast development. J. Biol. Chem..

[43] Eliopoulos AG, Wang CC, Dumitru CD, Tsichlis PN (2003). Tpl2 transduces CD40 and TNF signals that activate ERK and regulates IgE induction by CD40. EMBO J..

[44] Kobayashi T (2003). TRAF6 is a critical factor for dendritic cell maturation and development. Immunity.

[45] Hirai M (2017). Adaptor proteins NUMB and NUMBL promote cell cycle withdrawal by targeting ERBB2 for degradation. J. Clin. Invest..

[46] Zou J (2018). Neddylation mediates ventricular chamber maturation through repression of Hippo signaling. Proc. Natl Acad. Sci. USA.

[47] Wang Y (2010). Ephrin-B2 controls VEGF-induced angiogenesis and lymphangiogenesis. Nature.

[48] Heallen T (2013). Hippo signaling impedes adult heart regeneration. Development.

[49] Qu X, Harmelink C, Baldwin HS (2019). Tie2 regulates endocardial sprouting and myocardial trabeculation. JCI Insight.

[50] Wu Q (2020). Extracellular vesicles from human embryonic stem cell-derived cardiovascular progenitor cells promote cardiac infarct healing through reducing cardiomyocyte death and promoting angiogenesis. Cell Death Dis..

[51] Kim D, Langmead B, Salzberg SL (2015). HISAT: a fast spliced aligner with low memory requirements. Nat. Methods.

[52] Shen S (2014). rMATS: robust and flexible detection of differential alternative splicing from replicate RNA-Seq data. Proc. Natl Acad. Sci. USA.

