# Supplementary material for: Endothelial deletion of PTBP1 disrupts ventricular chamber development
Source: Nat Commun. 2023 Mar 31;14:1796. doi: 10.1038/s41467-023-37409-9 (PMC10066379; doi:10.1038/s41467-023-37409-9)
Supplement: Supplementary file 1 — Supplementary Information [file 41467_2023_37409_MOESM1_ESM.pdf]

SUPPLEMENTARY INFORMATION

Endothelial deletion of PTBP1 disrupts ventricular chamber development

Hongyu Liu, Ran Duan, Xiaoyu He, Jincu Qi, Tianming Xing, Yahan Wu, Liping Zhou, Lingling Wang, Yujing Shao, Fulei Zhang, Huixing Zhou, Xingdong Gu, Bowen Lin, Yuanyuan Liu, Yan Wang, Yi Liu, Li Li, Dandan Liang, and Yi-Han Chen

Table of contents:

Supplementary Fig. 1

Supplementary Fig. 2

Supplementary Fig. 3

Supplementary Fig. 4

Supplementary Fig. 5

Supplementary Fig. 6

Supplementary Fig. 7

Supplementary Fig. 8

Supplementary Fig. 9

Supplementary Fig. 10

Supplementary Fig. 11

Supplementary Fig. 12

Supplementary Fig. 13

Supplementary Fig. 14

Supplementary Fig. 15

Supplementary Table 1

Supplementary Table 2

Supplementary Table 3

Supplementary Table 4

Supplementary Fig. 16 (Uncropped images from Western blots)

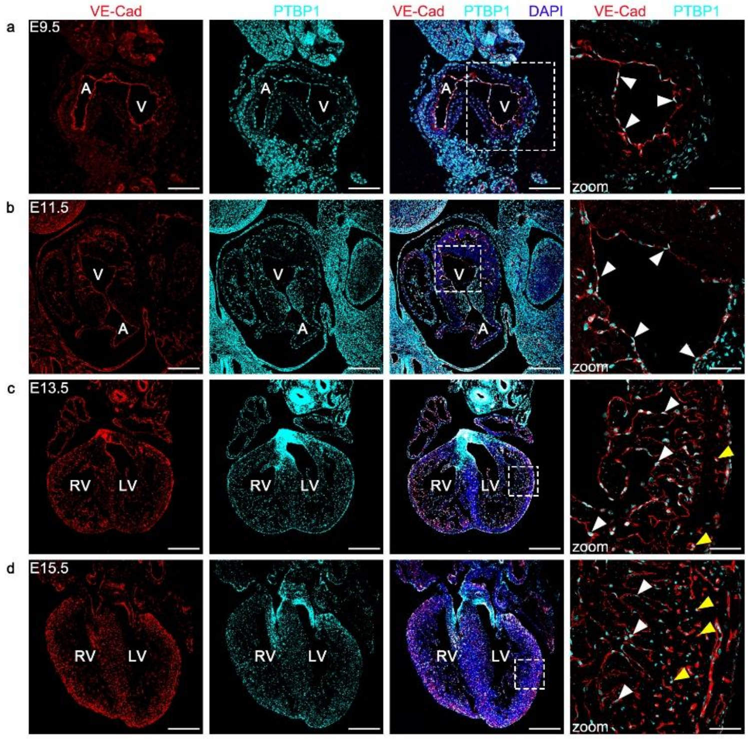

Supplementary Fig. 1. The colocalization of PTBP1 with endothelial cells during embryonic heart development. a-d Immunofluorescence showing the co-staining of PTBP1 (cyan) and the endothelial cell marker VE-cadherin (red) in embryonic hearts. The white arrowheads in the magnified images indicate the co-staining of PTBP1 with endocardial cells; the yellow arrowheads indicate the co-staining of PTBP1 with endothelial cells in compact myocardium. Scale bars, 100 μm in **a**; 200μm in **b**; 300 μm in **c**, **d**; 50 μm in the zoomed pictures. A, atrium; V, ventricle; LV, left ventricle; RV, right ventricle; VE-Cad, VE-Cadherin.

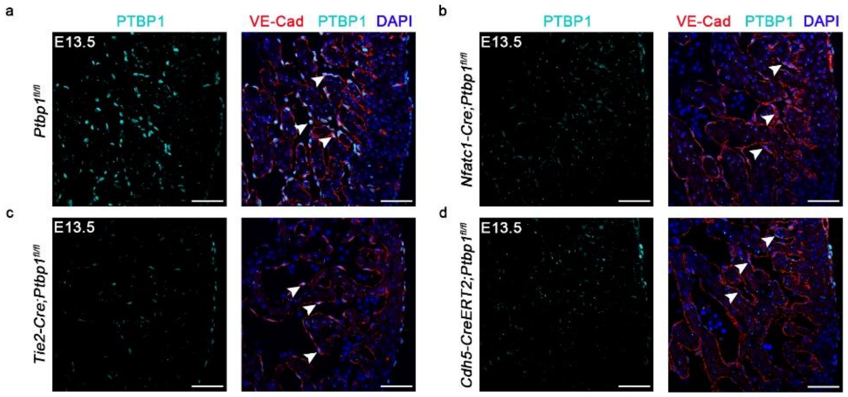

Supplementary Fig. 2. The expression of PTBP1 is significantly reduced in cardiac endothelial cells of *Ptbp1*-deficient mice. a-d Co-immunostaining of PTBP1 (cyan) and the endothelial cell marker VE-cadherin (red) in heart sections fro E13.5 *Ptbp1*$^{fl/fl}$ (**a**), *Nfatc1-Cre*;*Ptbp1*$^{fl/fl}$ (**b**), *Tie2-Cre*;*Ptbp1*$^{fl/fl}$ (**c**) and *Cdh5-CreERT2*;*Ptbp1*$^{fl/fl}$ (**d**) mice. The white arrowheads indicate PTBP1 staining in endothelial cells. VE-Cad, VE-Cadherin. Scale bars, 50 μm.

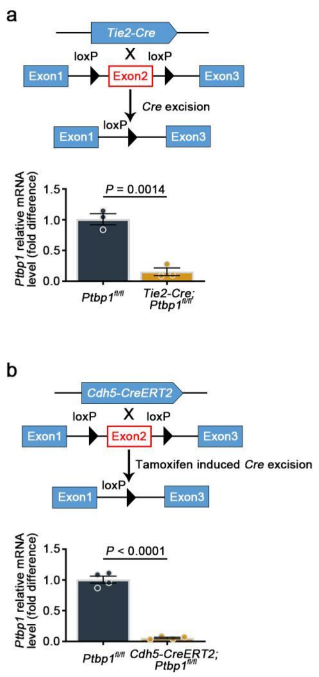

Supplementary Fig. 3. The significantly reduced expression of *Ptbp1* mRNA in pan-endothelial-specific knockout mice. a *Top*, schematic showing the generation of *Tie2-Cre*;$Ptbp1^{fl/fl}$ mice. *Bottom,* qRT-PCR assay showing the mRNA expression of *Ptbp1* in endothelial cells isolated from E13.5 $Ptbp1^{fl/fl}$ and *Tie2-Cre*;$Ptbp1^{fl/fl}$ hearts. n = 3 mice per group. **b** *Top*, schematic showing the generation of *Cdh5-CreERT2*;$Ptbp1^{fl/fl}$ mice. *Bottom,* qRT-PCR assay showing the mRNA expression of the *Ptbp1* in endothelial cells isolated from E13.5 $Ptbp1^{fl/fl}$ and *Cdh5-CreERT2*;$Ptbp1^{fl/fl}$ hearts. n = 4 mice per group. The data are presented as the mean ± s.e.m. *P* values were calculated by unpaired two-tailed t-test. Source data are provided as a Source Data file.

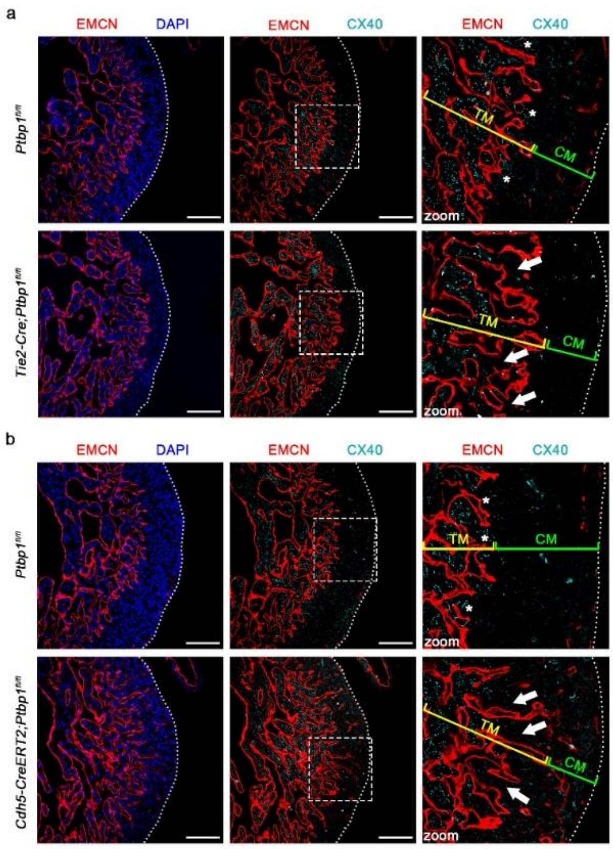

Supplementary Fig. 4. Abnormal trabecular myocardium in *Tie2-Cre*;*Ptbp1$^{fl/fl}$* and *Cdh5-CreERT2*;*Ptbp1$^{fl/fl}$* hearts. a Immunostaining of endomucin (EMCN) and CX40 in heart sections from E13.5 *Ptbp1$^{fl/fl}$* and *Tie2-Cre*;*Ptbp1$^{fl/fl}$* mice. Scale bars, 100 µm. **b** Immunostaining of EMCN and CX40 in heart sections from E15.5 *Ptbp1$^{fl/fl}$* and *Cdh5-CreERT2*;*Ptbp1$^{fl/fl}$* mice. Scale bars, 100 µm. The white lines indicate the heart border. TM, trabecular myocardium; CM, compact myocardium. The asterisks indicate the expression of CX40 in the *Ptbp1$^{fl/fl}$* hearts. The arrows indicate the reduced expression of CX40 in *Ptbp1*-deficient hearts.

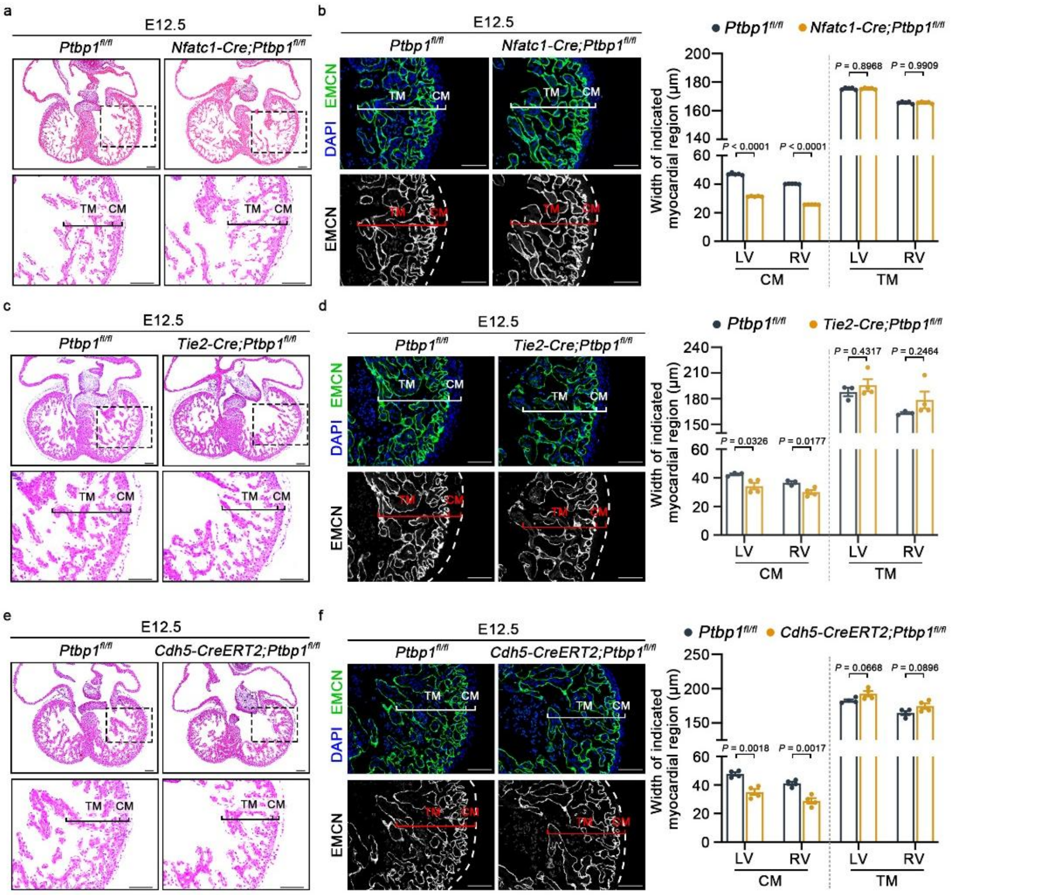

Supplementary Fig. 5. *Ptbp1* knockout in endothelial cells reduces the thickness of compact myocardium at E12.5. a-f H&E staining and immunofluorescence staining of endomucin (EMCN) showed the thinned compact myocardium (CM) in E12.5 *Nfatc1-Cre*;*Ptbp1$^{fl/fl}$* (**a, b**), *Tie2-Cre*;*Ptbp1$^{fl/fl}$* (**c, d**) and *Cdh5-CreERT2*;*Ptbp1$^{fl/fl}$* (**e, f**) mice compared to *Ptbp1$^{fl/fl}$* mice. Scale bars, 100 μm. The graphs showing the quantification of the CM and trabecular myocardium (TM) thickness in different groups. n = 5 mice per group (**b**), n = 3 mice for *Ptbp1$^{fl/fl}$* group and n= 4 mice for *Tie2-Cre*;*Ptbp1$^{fl/fl}$* group (**d**), n = 4 mice per group (**f**). The data are presented as the mean ± s.e.m. *P* values were calculated by unpaired two-tailed t-test. Source data are provided as a Source Data file.

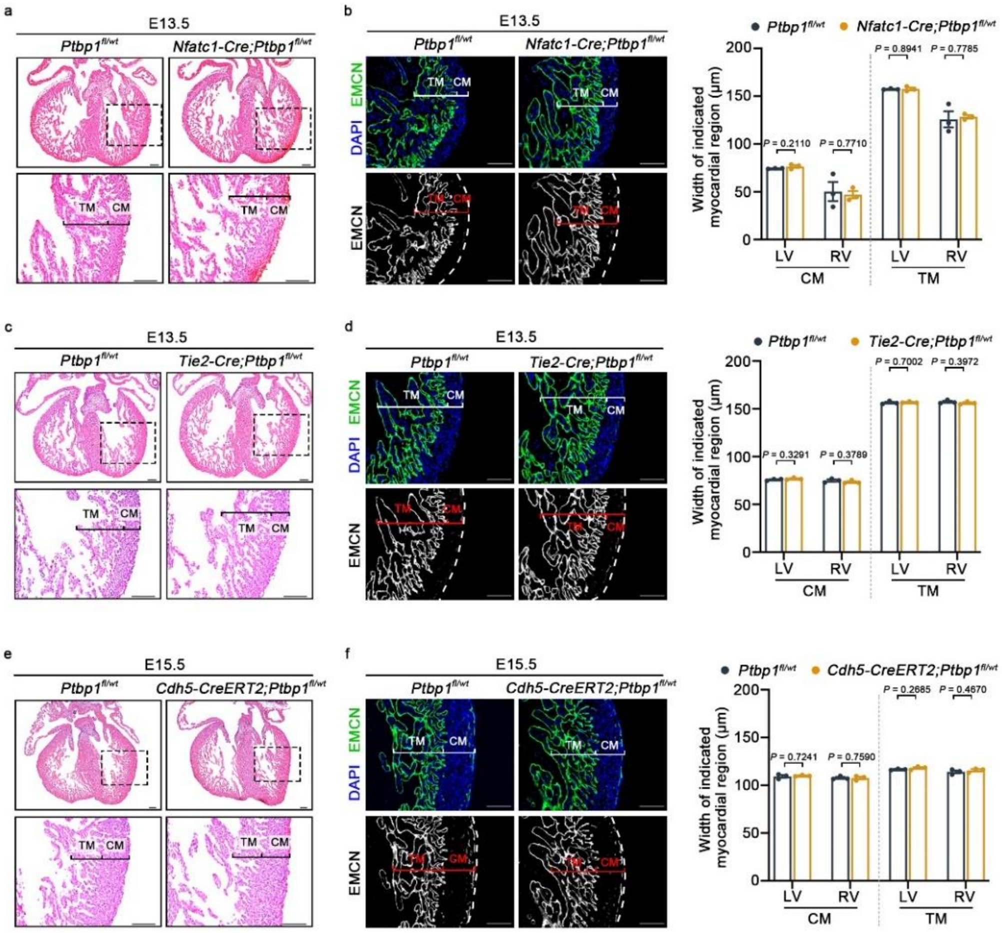

Supplementary Fig. 6. Heterozygous *Ptbp1*-deficient mice display normal ventricular morphology and structure. a-f H&E staining and Immunofluorescence staining for endomucin (EMCN) in heart sections showing the trabecular myocardium (TM) and compact myocardium (CM) in E13.5 *Nfatc1-Cre*;*Ptbp1$^{fl/wt}$* (**a, b**), E13.5 *Tie2-Cre*;*Ptbp1$^{fl/wt}$* (**c, d**) and E15.5 *Cdh5-CreERT2*;*Ptbp1$^{fl/wt}$* (**e, f**) mice. Scale bars, 100 μm. The graphs showing the quantification of the CM and TM thickness. n = 3 mice per group. The data are presented as the mean ± s.e.m. *P* values were calculated by unpaired two-tailed t-test. Source data are provided as a Source Data file.

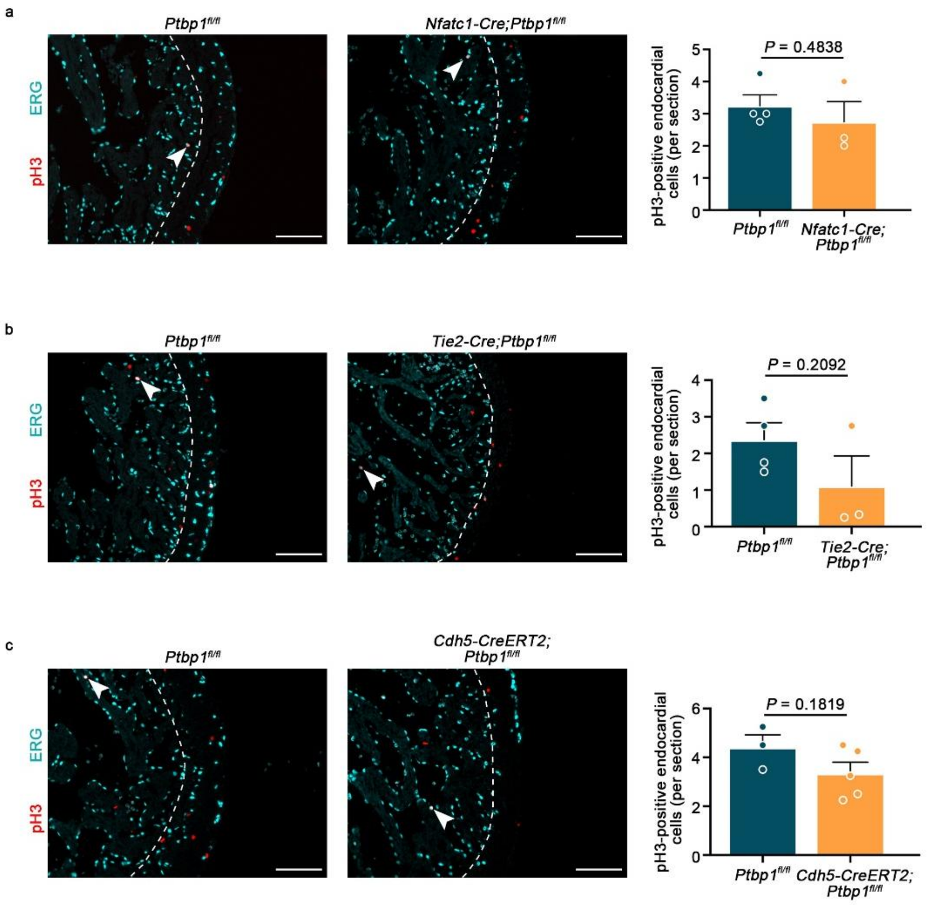

Supplementary Fig. 7. *Ptbp1* deficiency does not significantly change the proliferation of endocardial cells in mice. a-c Co-immunostaining of the proliferation marker pH3 (red) and the endothelial cell marker ERG (cyan) showing the proliferation of endocardial cells in heart sections from E13.5 *Nfatc1-Cre*;*Ptbp1$^{fl/fl}$* (**a**), *Tie2-Cre*;*Ptbp1$^{fl/fl}$* (**b**) and *Cdh5-CreERT2*;*Ptbp1$^{fl/fl}$* (**c**) mice, respectively. The white arrowheads indicate the pH3-positive endocardial cells. The white dashed lines indicate the border between compact myocardium and trabecular myocardium. Scale bars, 100 μm. The graphs showing the quantification of pH3-positive endocardial cells in trabecular myocardium of different groups. n = 4 mice for *Ptbp1$^{fl/fl}$* group and n = 3 mice for *Nfatc1-Cre*;*Ptbp1$^{fl/fl}$* group (**a**), n = 4 mice for *Ptbp1$^{fl/fl}$* group and n = 3 mice for *Tie2-Cre*;*Ptbp1$^{fl/fl}$* group (**b**), n = 3 mice for *Ptbp1$^{fl/fl}$* group and n = 5 mice for *Cdh5-CreERT2*;*Ptbp1$^{fl/fl}$* group (**c**) . Data are presented as the mean ± s.e.m. *P* values were calculated by unpaired two-tailed t-test. Source data are provided as a Source Data file.

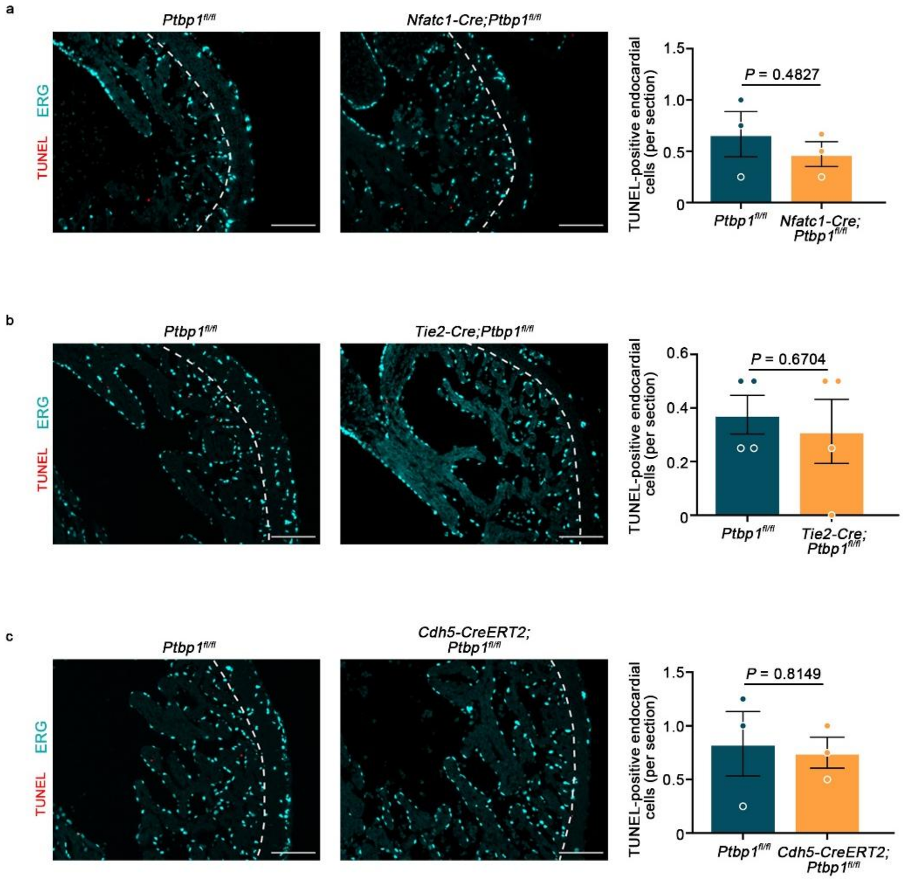

Supplementary Fig. 8. The apoptosis of endocardial cells is not significantly changed in *Ptbp1* knockout mice. a-c Co-immunostaining of the apoptosis marker TUNEL (red) and the endothelial cell marker ERG (cyan) showing the apoptosis of endocardial cells in heart sections from E13.5 *Nfatc1-Cre*;*Ptbp1*$^{fl/fl}$ (**a**), *Tie2-Cre*;*Ptbp1*$^{fl/fl}$ (**b**) and *Cdh5-CreERT2*;*Ptbp1*$^{fl/fl}$ (**c**) mice, respectively. The white dashed lines indicate the border between compact myocardium and trabecular myocardium. Scale bars, 100 μm. The graphs showing the quantification of TUNEL-positive endocardial cells in trabecular myocardium of different groups. n = 3 mice per group (**a**), n = 4 mice per group (**b**), n = 3 mice per group (**c**). Data are presented as the mean ± s.e.m. *P* values were calculated by unpaired two-tailed t-test. Source data are provided as a Source Data file.

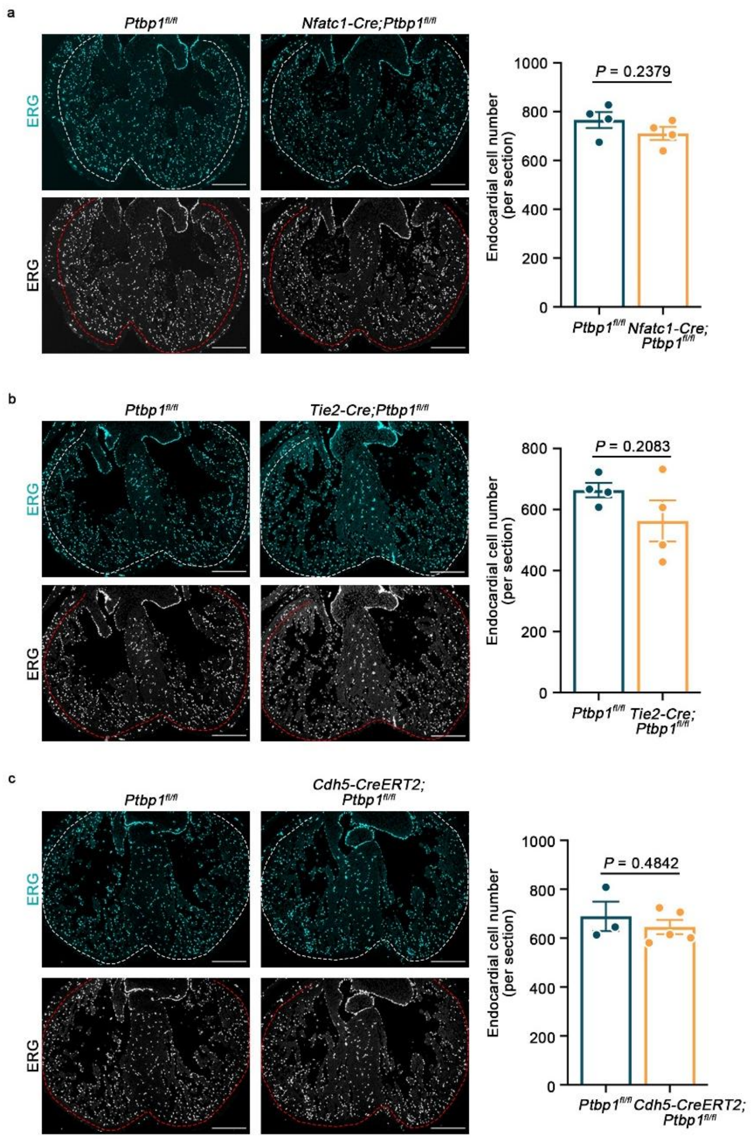

Supplementary Fig. 9. The number of endocardial cells is not changed after *Ptbp1* knockout in mice. a-c Immunostaining of ERG (cyan) showing the endothelial cells in heart sections from E13.5 *Nfatc1-Cre*;*Ptbp1$^{fl/fl}$* (**a**), *Tie2-Cre*;*Ptbp1$^{fl/fl}$* (**b**) and *Cdh5-CreERT2*;*Ptbp1$^{fl/fl}$* (**c**) mice, respectively. Scale bars, 200 µm. The white dashed lines indicate the border between compact myocardium and trabecular myocardium. The graphs showing the quantification of endocardial cell number in trabecular myocardium of different groups. n = 4 mice per group (**a**), n = 4 mice per group (**b**), n = 3 mice for *Ptbp1$^{fl/fl}$* group and n = 5 mice for *Cdh5-CreERT2*;*Ptbp1$^{fl/fl}$* group (**c**). Data are presented as the mean ± s.e.m. *P* values were calculated by unpaired two-tailed t-test. Source data are provided as a Source Data file.

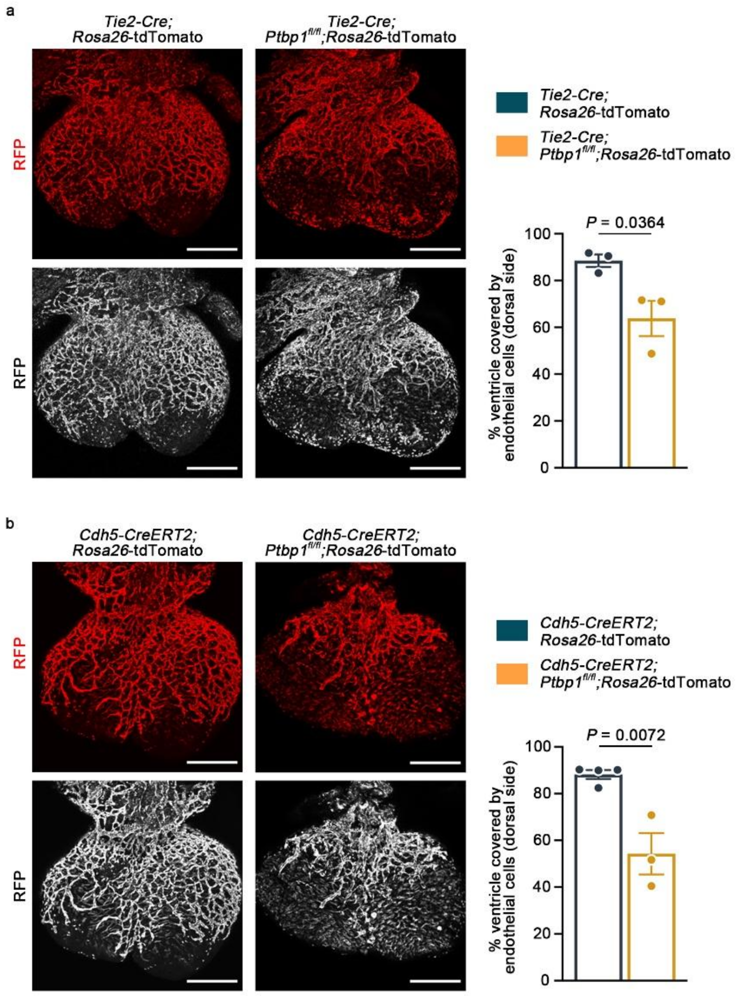

Supplementary Fig. 10. The growth of coronary vessel is suppressed after *Ptbp1* deletion in endothelial cells. a Whole-mount confocal images and quantification of ventricular area covered by endothelial cells on the dorsal side of the hearts in E13.5 *Tie2-Cre*;*Ptbp1*$^{fl/fl}$;*Rosa26*-tdTomato and *Tie2-Cre*;*Rosa26*-tdTomato mice. Scale bars, 200 μm. n = 3 mice per group. **b** Whole-mount confocal images and quantification of ventricular area covered by endothelial cells on the dorsal side of the hearts in E13.5 *Cdh5-CreERT2*;*Ptbp1*$^{fl/fl}$;*Rosa26*-tdTomato and *Cdh5-CreERT2*;*Rosa26*-tdTomato mice. Scale bars, 200 μm. n = 4 mice for *Cdh5-CreERT2*;*Rosa26*-tdTomato group and n = 3 mice for *Cdh5-CreERT2*;*Ptbp1*$^{fl/fl}$;*Rosa26*-tdTomato group. The data are presented as the mean ± s.e.m. *P* values were calculated by unpaired two-tailed t-test. Source data are provided as a Source Data file.

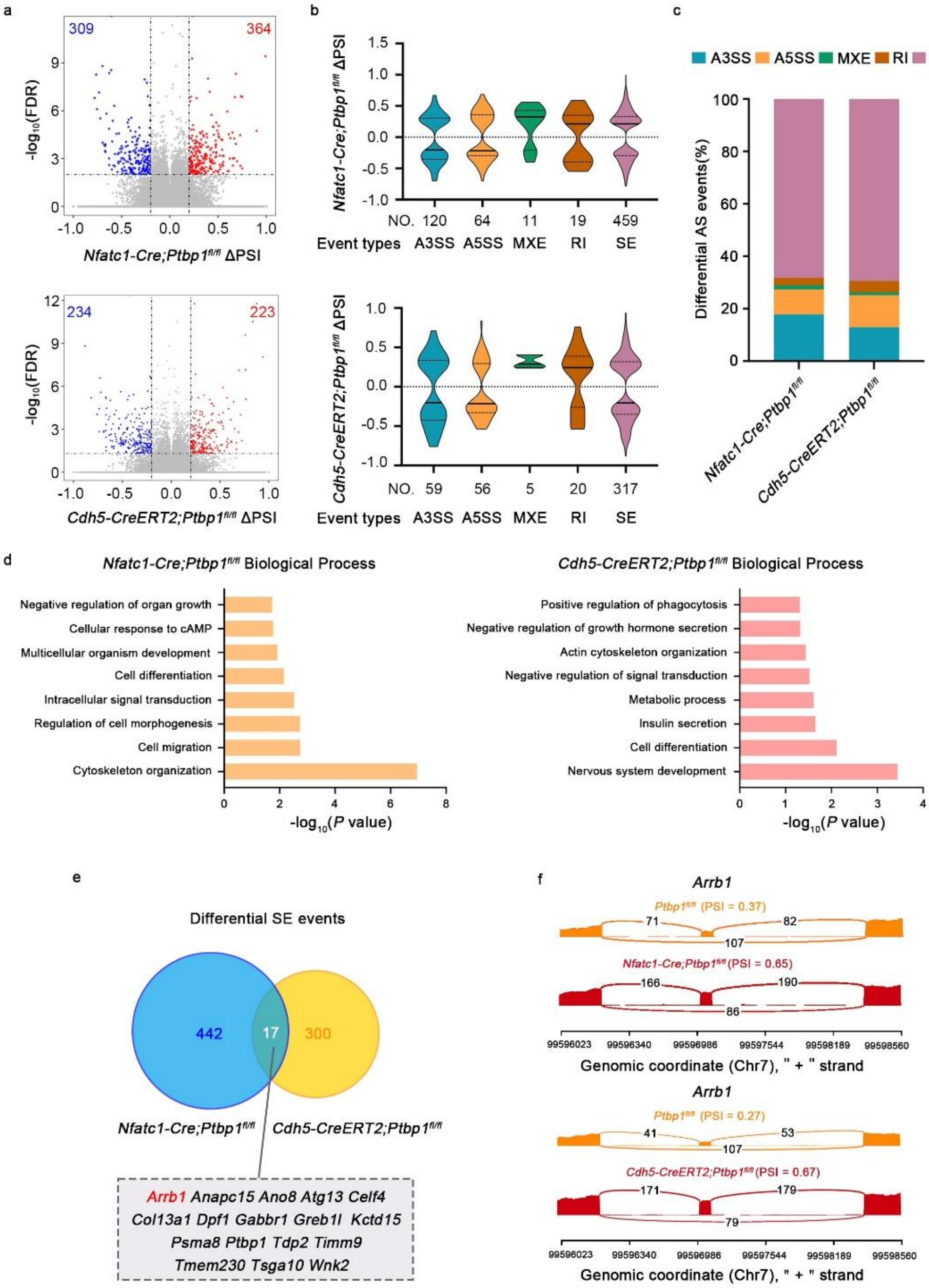

Supplementary Fig. 11. The alternative splicing analysis in *Ptbp1*-deficient cardiac endothelial cells. a Volcano plots indicate differential alternative splicing events identified in endothelial cells from E13.5 *Nfatc1-Cre*;*Ptbp1$^{fl/fl}$* or *Cdh5-CreERT2*;*Ptbp1$^{fl/fl}$* hearts compared to *Ptbp1$^{fl/fl}$* hearts, respectively. Significant alternative splicing events (FDR < 0.05 and |ΔPSI| ⩾ 0.2) are colored as blue or red dots if *Ptbp1* deletion resulted in skipping or inclusion, respectively. n = 3-4 mice per

group. PSI: Percent Spliced In. **b** Violin plots indicate the distribution of differential alternative splicing event types in endothelial cells from E13.5 *Nfatc1-Cre*;*Ptbp1*$^{fl/fl}$ or *Cdh5-CreERT2*;*Ptbp1*$^{fl/fl}$ hearts, respectively. **c** Stacked bar graph showing the proportions of differential alternative splicing events in different groups. **d** Gene Ontology (GO) enrichment analysis of genes with differential skipping exon (SE) events in different groups. GO analysis were performed to screen the significant enriched terms using R package clusterProfiler. **e** Venn diagram showing the common significant SE events between *Nfatc1-Cre*;*Ptbp1*$^{fl/fl}$ and *Cdh5-CreERT2*;*Ptbp1*$^{fl/fl}$ mice. The table showing the genes with common SE events. **f** Representative images of the Sashimi plots showing the exon splicing of *Arrb1*. The Sashimi plots show the densities of exon-including and exon-skipping reads as determined by rMATS-turbo analysis. A3SS: Alternative 3' Splice Site; A5SS: Alternative 5' Splice Site; MXE: Mutually Exclusive Exon; RI: Retention Intron; SE: Skipped Exon. Source data are provided as a Source Data file.

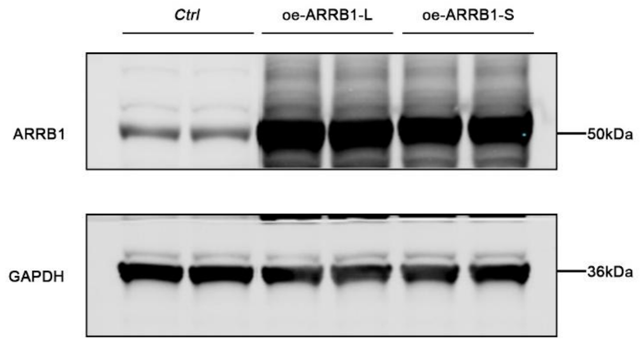

Supplementary Fig. 12. Western blot analysis confirms the overexpression efficiency of ARRB1-L and ARRB1-S plasmids in 293T cells.

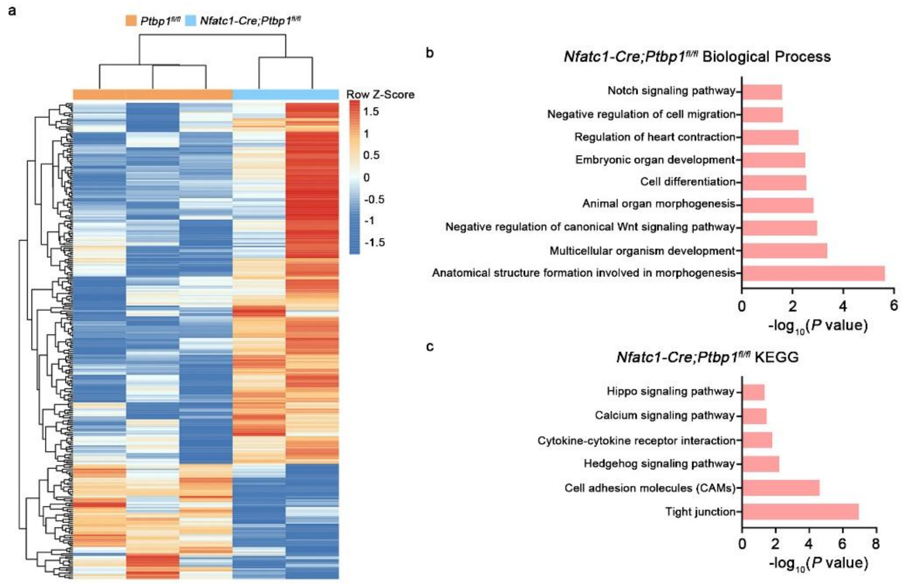

Supplementary Fig. 13. Analysis of differentially expressed genes (DEGs) in endothelial cells from *Ptbp1$^{fl/fl}$* and *Nfatc1-Cre*;*Ptbp1$^{fl/fl}$* hearts. a Heatmap showing the DEGs ($|\log_2 FC| > 0.58$ and $P < 0.05$) revealed by RNA-sequencing (RNA-seq) analysis in cardiac endothelial cells from E13.5 *Ptbp1$^{fl/fl}$* and *Nfatc1-Cre*;*Ptbp1$^{fl/fl}$* hearts. n = 2-3 mice per group. **b, c** Gene Ontology (GO) and Kyoto Encyclopedia of Genes and Genomes (KEGG) analysis of the DEGs in endothelial cells from E13.5 *Ptbp1$^{fl/fl}$* and *Nfatc1-Cre*;*Ptbp1$^{fl/fl}$* hearts. GO and KEGG enrichment analysis were performed to screen the significant enriched terms using R package clusterProfiler. Source data are provided as a Source Data file.

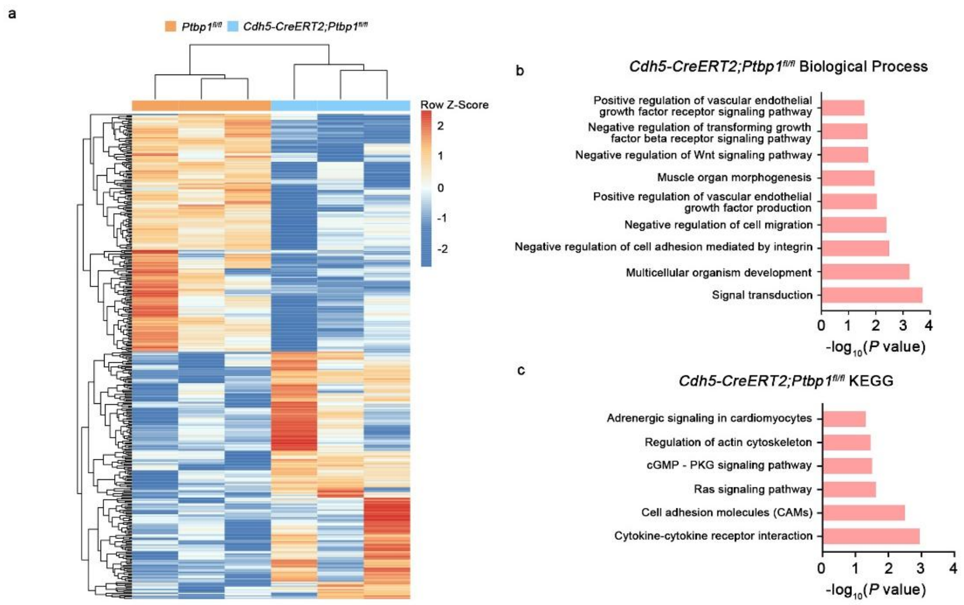

Supplementary Fig. 14. Analysis of differentially expressed genes (DEGs) in endothelial cells from *Ptbp1$^{fl/fl}$* and *Cdh5-CreERT2*;*Ptbp1$^{fl/fl}$* hearts. a Heatmap showing the DEGs (|$\log_2$ FC| > 0.58 and P < 0.05) revealed by RNA-sequencing (RNA-seq) analysis in cardiac endothelial cells from E13.5 *Ptbp1$^{fl/fl}$* and *Cdh5-CreERT2*;*Ptbp1$^{fl/fl}$* hearts. n = 3 mice per group. **b**, **c** Gene Ontology (GO) and Kyoto Encyclopedia of Genes and Genomes (KEGG) analysis of the DEGs in endothelial cells from E13.5 *Ptbp1$^{fl/fl}$* and *Cdh5-CreERT2*;*Ptbp1$^{fl/fl}$* hearts. GO and KEGG enrichment analysis were performed to screen the significant enriched terms using R package clusterProfiler. Source data are provided as a Source Data file.

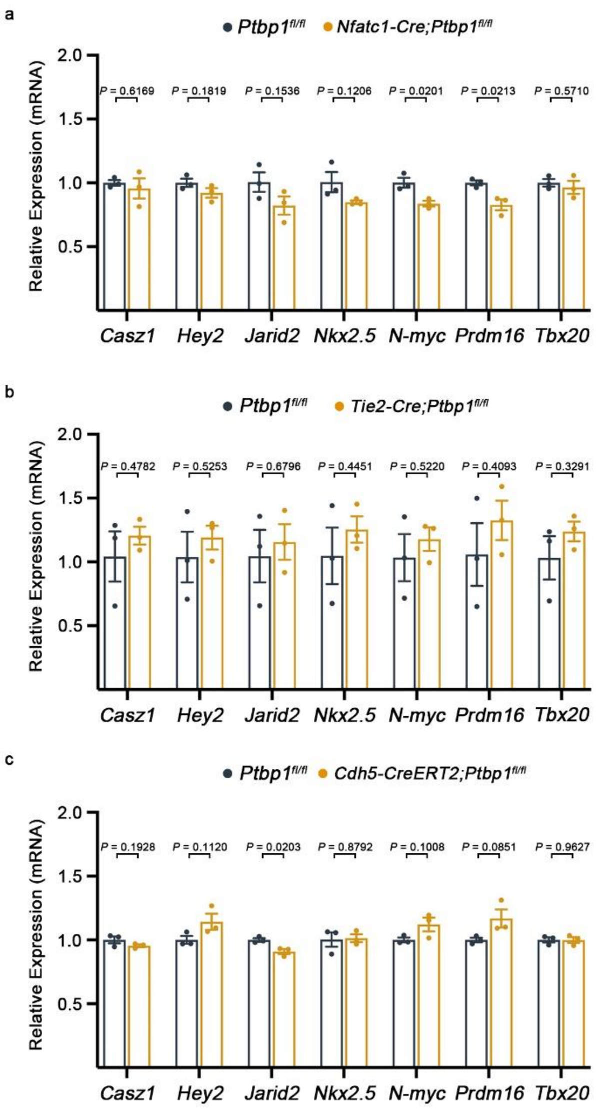

Supplementary Fig. 15. The expression of several LVNC-related transcription factors in *Ptbp1* knockout mice. a-c The graphs showing the mRNA expression of *Casz1, Hey2*, *Jarid2, Nkx2.5*, *N-myc*, *Prdm16* and *Tbx2*0 in E13.5 *Nfatc1-Cre*;*Ptbp1$^{fl/fl}$* (**a**), *Tie2-Cre*;*Ptbp1$^{fl/fl}$* (**b**) and *Cdh5-CreERT2*;*Ptbp1$^{fl/fl}$* (**c**) hearts compared to *Ptbp1$^{fl/fl}$* mice. n = 3 mice per group. The data are presented as the mean ± s.e.m. *P* values were calculated by two-tailed unpaired t-test. Source data are provided as a Source Data file.

Supplementary Table 1. Genotype frequency of offspring from *Ptbp1*$^{fl/fl}$ and *Nfatc1-Cre*;*Ptbp1*$^{fl/fl}$ interbreedings.

Age	Total Embryos	*Ptbp1*$^{fl/fl}$	*Nfatc1-Cre*;*Ptbp1*$^{fl/fl}$	*P* value
**Theoretical ratio**		50%	50%	
**Experimental ratio**				
E11.5	50	21 (42%)	29 (58%)	0.3207
E12.5	39	21 (54%)	18 (46%)	0.6712
E13.5	230	111 (48%)	119 (52%)	0.8876
Adult	115	53 (46%)	62 (54%)	0.6712

Chi-square tests were used to assess statistical significance for genotype ratios between experimental and theoretical values, and $P < 0.05$ was considered statistically significant.

Supplementary Table 2. Genotype frequency of offspring from *Ptbp1*$^{fl/fl}$ and *Cdh5-CreERT2*;*Ptbp1*$^{fl/fl}$ interbreedings.

Age	Total Embryos	*Ptbp1*$^{fl/fl}$	*Cdh5-CreERT2*;*Ptbp1*$^{fl/fl}$	*P* value
**Theoretical ratio**		50%	50%	
**Experimental ratio**				
E11.5	16	7 (44%)	9 (56%)	0.4788
E12.5	23	14 (61%)	9 (39%)	0.1546
E13.5	139	90 (65%)	49 (35%)	0.0449
E15.5	102	69 (68%)	33 (32%)	0.0143
E17.5	6	5 (83%)	1 (17%)	<0.0001

Chi-square tests were used to assess statistical significance for genotype ratios between experimental and theoretical values, and $P < 0.05$ was considered statistically significant.

Supplementary Table 3. Genotype frequency of offspring from *Ptbp1$^{fl/fl}$* and *Tie2-Cre*;*Ptbp1$^{fl/fl}$* interbreedings.

Age	Total Embryos	*Ptbp1$^{fl/fl}$*	*Tie2-Cre*;*Ptbp1$^{fl/fl}$*	*P* value
**Theoretical ratio**		50%	50%	
**Experimental ratio**				
E11.5	21	11 (52%)	10 (48%)	0.8876
E12.5	36	19 (53%)	17 (47%)	0.7773
E13.5	56	37 (66%)	19 (34%)	0.0314
Adult	69	45 (65%)	24 (35%)	0.0449

Chi-square tests were used to assess statistical significance for genotype ratios between experimental and theoretical values, and $P < 0.05$ was considered statistically significant.

Supplementary Table 4. List of primers used for qRT-PCR.

Gene	Forward Primer	Reverse Primer
Mus-*Ptbp1*	TCCCAGACATAGCAGTCGGTACA	TGACATGGATGACTCTGGAAGG
Mus-*Nkx2.5*	TGCTCTCCTGCTTTCCCAGCC	CTTTGTCCAGCTCCACTGCCTT
Mus-*Casz1*	GCTCATCAACGATGGCTTCCAG	GTGGTAGCTCTTGTGCTTCTCG
Mus-*Jarid2*	GCCTGCATAAAGAAGCACCTCAG	GAGGTCAGTCACTTGCTGCATG
Mus-*Prdm16*	ATCCACAGCACGGTGAAGCCAT	ACATCTGCCCACAGTCCTTGCA
Mus-*Hey2*	GCTCATTGACACCAACTC	ATTGCCTGCTTCTTCTCT
Mus-*Tbx20*	TATTCAGCATACTCCTAC	GTTAGTCTTGTCAATACG
Mus-*N-myc*	GACGAGGAGGAAGATGAA	CTTGTTGTTAGAGGAGGAA
Mus-*Gapdh*	CGTGCCGCCTGGAGAAAC	AGTGGGAGTTGCTGTTGAAGTC

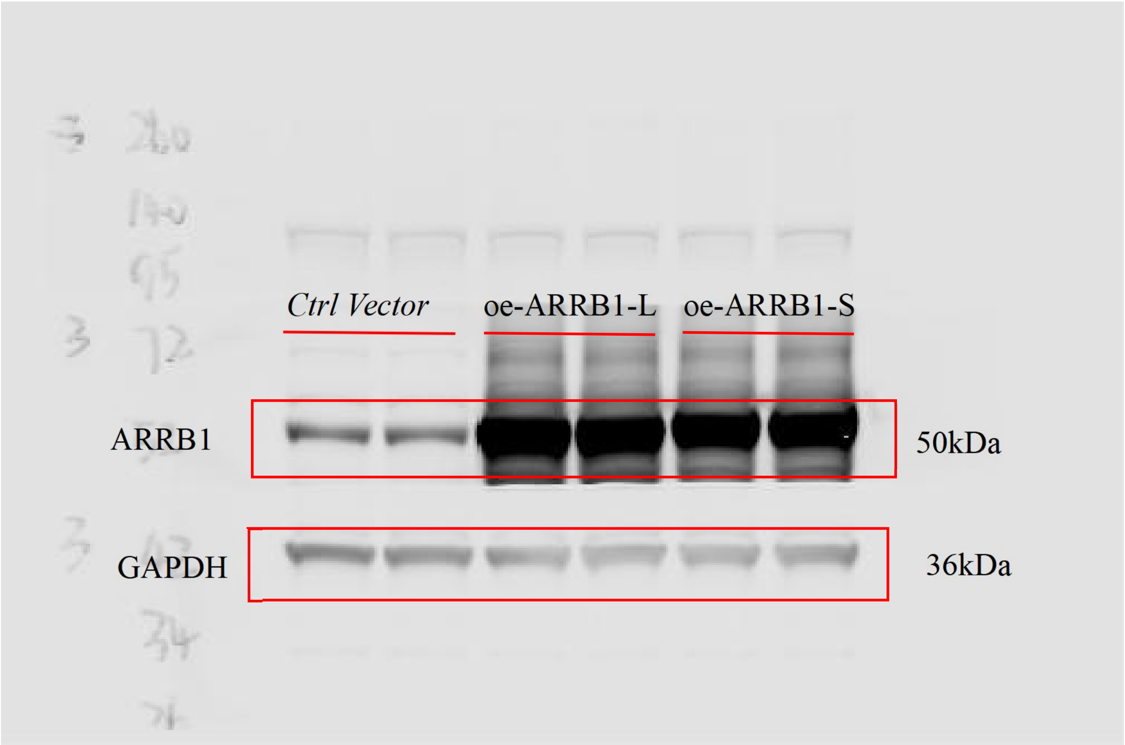

Supplementary Fig. 16 Uncropped images of Western blots in Supplementary Fig. 12.
