# Supplementary material for: Endothelial deletion of PTBP1 disrupts ventricular chamber development
Source: Nat Commun. 2023 Mar 31;14:1796. doi: 10.1038/s41467-023-37409-9 (PMC10066379; doi:10.1038/s41467-023-37409-9)
Supplement: Supplementary file 2 — Reporting Summary [file 41467_2023_37409_MOESM2_ESM.pdf]

nature portfolio

Corresponding author(s): Yi-Han Chen

Last updated by author(s): Mar 6, 2023

Reporting Summary

Nature Portfolio wishes to improve the reproducibility of the work that we publish. This form provides structure for consistency and transparency in reporting. For further information on Nature Portfolio policies, see our Editorial Policies and the Editorial Policy Checklist.

Statistics

For all statistical analyses, confirm that the following items are present in the figure legend, table legend, main text, or Methods section.

n/a	Confirmed	
☐	☒	The exact sample size (*n*) for each experimental group/condition, given as a discrete number and unit of measurement
☐	☒	A statement on whether measurements were taken from distinct samples or whether the same sample was measured repeatedly
☐	☒	The statistical test(s) used AND whether they are one- or two-sided<br>*Only common tests should be described solely by name; describe more complex techniques in the Methods section.*
☒	☐	A description of all covariates tested
☒	☐	A description of any assumptions or corrections, such as tests of normality and adjustment for multiple comparisons
☐	☒	A full description of the statistical parameters including central tendency (e.g. means) or other basic estimates (e.g. regression coefficient) AND variation (e.g. standard deviation) or associated estimates of uncertainty (e.g. confidence intervals)
☐	☒	For null hypothesis testing, the test statistic (e.g. *F*, *t*, *r*) with confidence intervals, effect sizes, degrees of freedom and *P* value noted<br>*Give P values as exact values whenever suitable.*
☒	☐	For Bayesian analysis, information on the choice of priors and Markov chain Monte Carlo settings
☒	☐	For hierarchical and complex designs, identification of the appropriate level for tests and full reporting of outcomes
☒	☐	Estimates of effect sizes (e.g. Cohen's *d*, Pearson's *r*), indicating how they were calculated

Our web collection on statistics for biologists contains articles on many of the points above.

Software and code

Policy information about availability of computer code

Data collection	Histology, immunofluorescence and migration assays: Leica DMi8 microscope.<br>Whole-mount immunofluorescence, explant immunostaining and immunofluorescence images: Leica TCS SP8 and Leica DMi8 microscope.<br>RNA-seq: Illumina HiSeq X10 system.<br>qRT-PCR: StepOnePlus Real-Time PCR system (Applied Biosystems).<br>Gel electrophoresis and western blot: ChemiDoc™ Imaging Systems (Bio-Rad).
Data analysis	Statistical data were analyzed using GraphPad Prism 8.0; sequencing reads were separately aligned HISAT2; alternative splicing analysis was conducted using rMATS (version 4.1.0); GO and KEGG enrichment analysis of were performed to screen the significant enriched term using R package clusterProfiler respectively; immunofluorescent images, gel electrophoresis band density and migration assays images were analyzed using ImageJ software(1.5K).

For manuscripts utilizing custom algorithms or software that are central to the research but not yet described in published literature, software must be made available to editors and reviewers. We strongly encourage code deposition in a community repository (e.g. GitHub). See the Nature Portfolio guidelines for submitting code & software for further information.

Data

Policy information about availability of data

All manuscripts must include a data availability statement. This statement should provide the following information, where applicable:

- Accession codes, unique identifiers, or web links for publicly available datasets
- A description of any restrictions on data availability
- For clinical datasets or third party data, please ensure that the statement adheres to our policy

Data supporting the findings of this study are available from the authors on reasonable request. The data supporting the findings of this study are available within the article and Supplementary Information files. Source data are provided with this paper. The RNA-seq data have been deposited in the NCBI Sequence Read Archive under accession number PRJNA772292 (https://www.ncbi.nlm.nih.gov/sra/?term=PRJNA772292) and PRJNA862199 (https://www.ncbi.nlm.nih.gov/sra/?term=PRJNA862199). Figures associated with the RNA-seq data are Figure 6, Figure 7, Supplementary Figure 11, Supplementary Figure 12, Supplementary Figure 13 and Supplementary Figure 14. The accessible link for GRCm38.p6 database used in this study is https://www.gencodegenes.org/mouse/release_M20.html.

Human research participants

Policy information about studies involving human research participants and Sex and Gender in Research.

Reporting on sex and gender	N/A
Population characteristics	N/A
Recruitment	N/A
Ethics oversight	N/A

Note that full information on the approval of the study protocol must also be provided in the manuscript.

Field-specific reporting

Please select the one below that is the best fit for your research. If you are not sure, read the appropriate sections before making your selection.

☒ Life sciences ☐ Behavioural & social sciences ☐ Ecological, evolutionary & environmental sciences

For a reference copy of the document with all sections, see nature.com/documents/nr-reporting-summary-flat.pdf

Life sciences study design

All studies must disclose on these points even when the disclosure is negative.

Sample size	No statistical methods were used to predetermine sample size estimates. Sample size was determined based on standards in the field and experiments to obtain statistical significance and reproducibility. At least triplicates were used to meet the minimal requirements for statistical analysis and the detailed sample size was demonstrated in the figure legends.
Data exclusions	No data was excluded from analysis.
Replication	To ensure the replication of the findings, experiments were repeated at different times as indicated in the figure legends. All experimental data was reliably reproduced in multiple independent experiments. For in vivo experiments, multiple mice were used to ensure reproducibility, the exact number of animals was shown in the figure legends.
Randomization	For in vivo experiments, experimental samples were determined by the genotypes of the mice. For migration assays, HUVECs were randomly assigned to the experimental group.
Blinding	For in vivo studies, genotype was not disclosed to the investigators performing data collection and those generating quantitative readouts. Experimenters were blinded to the group allocation during data collection and analysis.

Reporting for specific materials, systems and methods

We require information from authors about some types of materials, experimental systems and methods used in many studies. Here, indicate whether each material, system or method listed is relevant to your study. If you are not sure if a list item applies to your research, read the appropriate section before selecting a response.

Materials & experimental systems

n/a	Involved in the study
☐	☒ Antibodies
☐	☒ Eukaryotic cell lines
☒	☐ Palaeontology and archaeology
☐	☒ Animals and other organisms
☒	☐ Clinical data
☒	☐ Dual use research of concern

Methods

n/a	Involved in the study
☒	☐ ChIP-seq
☒	☐ Flow cytometry
☒	☐ MRI-based neuroimaging

Antibodies

Antibodies used

Primary antibodies used in Immunofluorescence experiments:
1.anti-cTnT antibody (Abcam, ab8295, Monoclonal, Mouse, 1:50 dilution)
2.anti-PTBP1 antibody (Abcam, ab133734, Monoclonal, Rabbit, 1:100 dilution)
3.anti-RFP antibody (Abcam, ab124754, Polyclonal, Rabbit, 1:200 dilution)
4.anti-PTBP1 antibody (Sigma, WH0005725M1, Monoclonal, Mouse, 1:150 dilution)
5.anti-EMCN antibody (Santa Cruz, sc-65495, Monoclonal, Rat, 1:200 dilution)
6.anti-CX40 antibody (Thermo, 37-8900, Monoclonal, Mouse, 1:50 dilution)
7.anti-pH3 antibody (Abcam, ab32107, Monoclonal, Rabbit, 1:1000 dilution)
8.anti-NKX2.5 antibody (Abcam, ab97355, Polyclonal, Rabbit, 1:100 dilution)
9.anti-VE-Cadherin antibody (R&D Systems, AF1002-SP, Polyclonal, Goat, 1:100 dilution)
10.anti-ERG antibody (Abcam, ab92513, Monoclonal, Rabbit, 1:200 dilution)
11.anti-pH3 antibody (CST, 9706S, Monoclonal,Mouse, 1:100 dilution)

Primary antibodies used in Western Blots experiment:
1.anti-ARRB1 antibody (Abcam, ab32099, Monoclonal, Rabbit, 1:500 dilution)
2.anti-GAPDH antibody (Proteintech, 60004-1-Ig , Monoclonal, Mouse, 1:10000 dilution)

Secondary antibodies included:
1.Goat anti-Mouse IgG Alexa Fluor® 488: Abcam, ab150113, Goat, 1:200 dilution
2.Goat anti-Rabbit IgG Alexa Fluor® 555: Abcam, ab150078, Goat, 1:200 dilution
3.Goat anti-Mouse IgG Alexa Fluor® 555: Abcam, ab150118, Goat, 1:200 dilution
4.Goat anti-Rabbit IgG Alexa Fluor® 488: Abcam, ab150081, Goat, 1:200 dilution
5.Goat Anti-Rat IgG H&L (Alexa Fluor® 488) preadsorbed: Abcam, ab150165, Goat, 1:200 dilution
6.Donkey Anti-Goat IgG Alexa Fluor® 555: Abcam, ab150134, Donkey, 1:200 dilution
7.Goat anti-Mouse IgG (H+L) Highly Cross-Adsorbed Secondary Antibody, Alexa Fluor Plus 800: Invitrogen, A32730, Goat, 1:10000 dilution
8.Goat anti-Rabbit IgG (H+L) Highly Cross-Adsorbed Secondary Antibody, Alexa Fluor Plus 800: Invitrogen, A32735, Goat, 1:10000 dilution

Validation

All following primary antibodies used in this study were validated or cited in the litterature.
1. cTnT: https://www.abcam.com/cardiac-troponin-t-antibody-1c11-ab8295.html
Ref: Wu, Y. et al. LRP6 downregulation promotes cardiomyocyte proliferation and heart regeneration. Cell Res 31, 450-462 (2021).
2. PTBP1: https://www.abcam.com/ptbp1-antibody-epr9048b-ab133734.html
3. RFP: https://www.abcam.com/rfp-antibody-ab124754.html
Ref: Liu, X. et al. Cell-Type-Specific Interleukin 1 Receptor 1 Signaling in the Brain Regulates Distinct Neuroimmune Activities. Immunity 50, 764-766 (2019).
4. PTBP1: https://www.sigmaaldrich.cn/CN/zh/product/sigma/wh0005725m1?context=product
5. EMCN: https://www.scbt.com/p/endomucin-antibody-v-7c7
Ref: Rhee, S. et al. Endothelial deletion of Ino80 disrupts coronary angiogenesis and causes congenital heart disease. Nat Commun 9, 368 (2018).
6. CX40: https://www.thermofisher.cn/cn/zh/antibody/product/Connexin-40-Antibody-clone-2F9A11-Monoclonal/37-8900?adobe_mc=MCMID%7C62773349538989254870855660139045898647%7CMCAID%3D2E9D7E3585310FFD-4000010E00007C80%7CMCORGID%3D5B135A0C5370E6B40A490D44%40AdobeOrg%7CTS=1614293705
Ref: Boittin, F.X. et al. Connexins and M3 muscarinic receptors contribute to heterogeneous Ca(2+) signaling in mouse aortic endothelium. Cell Physiol Biochem 31, 166-178 (2013).
7. pH3: https://www.abcam.com/histone-h3-phospho-s10--t11-antibody-e173-ab32107.html
Ref: Wu, Y. et al. LRP6 downregulation promotes cardiomyocyte proliferation and heart regeneration. Cell Res 31, 450-462 (2021).
8. NKX2.5: https://www.abcam.com/nkx25-antibody-ab97355.html
Ref: Hu, W. et al. Smad4 regulates the nuclear translocation of Nkx2-5 in cardiac differentiation. Sci Rep 11, 3588 (2021).
9. VE-Cadherin: https://www.rndsystems.com/cn/products/mouse-ve-cadherin-antibody_af1002
Ref: Hong, S.P. et al. Distinct fibroblast subsets regulate lacteal integrity through YAP/TAZ-induced VEGF-C in intestinal villi. Nat Commun 11, 4102 (2020).
10.ERG: https://www.abcam.com/erg-antibody-epr3864-ab92513.html
11.pH3: https://www.cellsignal.com/products/primary-antibodies/phospho-histone-h3-ser10-6g3-mouse-mab/9706
Ref: Miyamoto K, Kawakami K, Tamura K, Abe G. Developmental independence of median fins from the larval fin fold revises their evolutionary origin. Sci Rep 12, 7521 (2022).
12.ARRB1: https://www.abcam.com/nav/primary-antibodies/rabbit-monoclonal-antibodies/beta-arrestin-1-antibody-e274-ab32099.html
Ref: Zhang, X. et al. ARRB1 Drives Gallbladder Cancer Progression by Facilitating TAK1/MAPK Signaling Activation. J Cancer 12,

1926-1935 (2021).
13.GAPDH:https://www.ptglab.com/products/GAPDH-Antibody-60004-1-Ig.htm
Ref: Liu, M. et al. Sox17 is required for endothelial regeneration following inflammation-induced vascular injury. Nat Commun 10, 2126 (2019).

Eukaryotic cell lines

Policy information about cell lines and Sex and Gender in Research

Cell line source(s)	Primary human umbilical endothelial cells (HUVECs) were purchased from PromoCell (C-12208) and HEK 293T cells were purchased from Shanghai Zhong Qiao Xin Zhou Biotechnology Co.,Ltd.(ZQ0033).
Authentication	Primary human umbilical endothelial cells (HUVECs) and HEK 293T cells were authenticated by the manufacturer. No further validation was performed.
Mycoplasma contamination	We have confirmed that the cells were tested negative for mycoplasma contamination.
Commonly misidentified lines (See ICLAC register)	We did not use a commonly misidentified cell line in this study.

Animals and other research organisms

Policy information about studies involving animals; ARRIVE guidelines recommended for reporting animal research, and Sex and Gender in Research

Laboratory animals	All mice were maintained on a mixed C57BL6 genetic background. An endocardial-specific Ptbp1 deficient mouse line was generated by crossing the Ptbp1fl/fl mice with Nfatc1-Cre mice. The pan-endothelial-specific Ptbp1 deficient mouse line was generated by crossing the Ptbp1fl/fl mice with Tie2-Cre or Cdh5-CreERT2 mice, respectively. To trace endothelial lineages, Rosa26-tdTomato mice were crossed with Tie2-cre, Nfatc1-Cre and Cdh5-CreERT2 mice. Age of mice operated and used for experiments were E9.5, E11.5, E12.5, E13.5, E15.5, E17.5 mice, and Adult mice.
Wild animals	Not involved.
Reporting on sex	Genders were not considered when analyzing the cardiac phenotypes of embryos.
Field-collected samples	Not involved.
Ethics oversight	All procedures involving animals conformed to the Guidelines for the Care and Use of Laboratory Animals established by the U.S. National Institutes of Health (National Academies Press; 2011) and were approved by Tongji University School of Medicine.

Note that full information on the approval of the study protocol must also be provided in the manuscript.
